# A predictive endocrine resistance index accurately stratifies luminal breast cancer treatment responders and nonresponders

**DOI:** 10.1172/JCI177813

**Published:** 2025-07-24

**Authors:** Guokun Zhang, Vindi Jurinovic, Stephan Bartels, Matthias Christgen, Henriette Christgen, Leonie Donata Kandt, Lidiya Mishieva, Hua Ni, Mieke Raap, Janin Klein, Anna-Lena Katzke, Winfried Hofmann, Doris Steinemann, Ronald E. Kates, Oleg Gluz, Monika Graeser, Sherko Kümmel, Ulrike Nitz, Christoph Plass, Ulrich Lehmann, Christine zu Eulenburg, Ulrich Mansmann, Clarissa Gerhäuser, Nadia Harbeck, Hans H. Kreipe

**Affiliations:** 1 Institute for Medical Information Processing, Biometry, and Epidemiology, Medical Faculty, Ludwig Maximilians University (LMU), Munich, Germany.; 2 Institute of Pathology, Hannover Medical School, Hannover, Germany.; 3 Institute of Sociology (IfS), Faculty of Social Sciences, University of Bremen, Bremen, Germany.; 4 Department of Methodology and Statistics, Faculty of Social & Behavioural Sciences, Utrecht University, Utrecht, Netherlands.; 5 Breast Center, Department OB&GYN and CCC Munich, LMU University Hospital, Munich, Germany.; 6 Department of Human Genetics, Hannover Medical School, Hannover, Germany.; 7 West German Study Group (WSG), Moenchengladbach, Germany.; 8 Ev. Bethesda Hospital, Breast Center Niederrhein, Moenchengladbach, Germany.; 9 University Clinics Cologne, Women’s Clinic and Breast Center, Cologne, Germany.; 10 Department of Gynecology, University Medical Center, Hamburg, Germany.; 11 Clinics Essen-Mitte, Breast Unit, Essen, Germany.; 12 Charité - Universitätsmedizin Berlin, Department of Gynecology with Breast Center, Berlin, Germany.; 13 Division of Cancer Epigenomics, German Cancer Research Center (DKFZ), Heidelberg, Germany.; 14 Department of Biometry and Epidemiology, University Medical Center Hamburg, Hamburg, Germany.

**Keywords:** Clinical Research, Clinical trials, Oncology, Bioinformatics, Breast cancer, Epigenetics

## Abstract

**BACKGROUND:**

Endocrine therapy (ET) with tamoxifen (TAM) or aromatase inhibitors (AI) is highly effective against hormone receptor–positive (HR-positive) early breast cancer (BC), but resistance remains a major challenge. The primary objectives of our study were to understand the underlying mechanisms of primary resistance and to identify potential biomarkers.

**METHODS:**

We selected more than 800 patients in 3 subcohorts (Discovery, *n* = 364, matched pairs; Validation 1, *n* = 270, Validation 2, *n* = 176) of the West German Study Group (WSG) ADAPT trial who underwent short-term preoperative TAM or AI treatment. Treatment response was assessed by immunohistochemical labeling of proliferating cells with Ki67 before and after ET. We performed comprehensive molecular profiling, including targeted next-generation sequencing (NGS) and DNA methylation analysis using EPIC arrays, on posttreatment tumor samples.

**RESULTS:**

*TP53* mutations were strongly associated with primary resistance to both TAM and AI. We identified distinct DNA methylation patterns in resistant tumors, suggesting alterations in key signaling pathways and tumor microenvironment composition. Based on these findings and patient age, we developed the Predictive Endocrine ResistanCe Index (PERCI). PERCI accurately stratified responders and nonresponders in both treatment groups in all 3 subcohorts and predicted progression-free survival in an external validation cohort and in the combined subcohorts.

**CONCLUSION:**

Our results highlight the potential of PERCI to guide personalized endocrine therapy and improve patient outcomes.

**TRIAL REGISTRATION:**

WSG-ADAPT, ClinicalTrials.gov NCT01779206, retrospectively registered 01-25-2013.

**FUNDING:**

German Cancer Aid (Grant Number 70112954), German Federal Ministry of Education and Research (Grant Number 01ZZ1804C, DIFUTURE).

## Abstract 2

Large short-term intervention study identifies epi-/genetic features associated with primary resistance to tamoxifen and aromatase inhibitors in HR+/HER2- breast cancer and develops accurate predictors.

## Graphical abstract

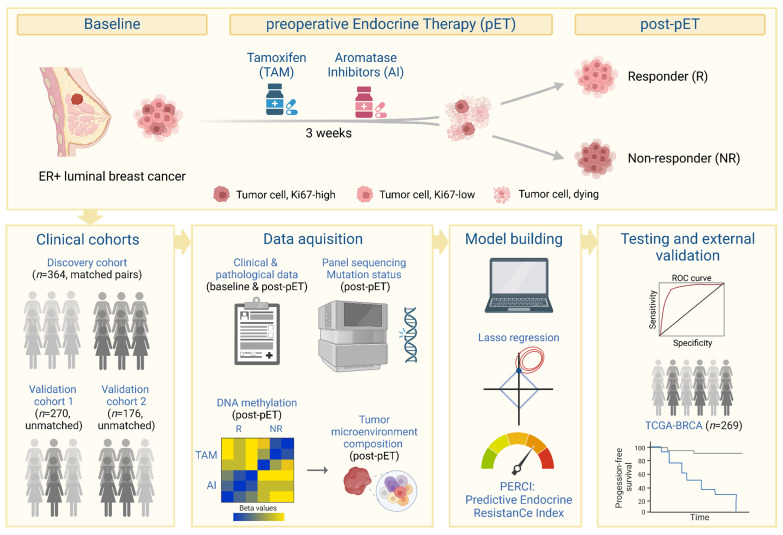

## Introduction

Endocrine therapy (ET) is highly effective in blocking estrogen receptor (ER) signaling in breast cancer (BC). However, up to 40% of patients diagnosed with operable ER-positive and/or progesterone receptor–positive (PR-positive), human epidermal growth factor receptor 2–negative (HER2-negative) (i.e. luminal) tumors relapse during or after adjuvant ET (1). Whereas the mechanisms of primary, intrinsic resistance remain incompletely understood, the mechanisms underlying secondary, acquired resistance are frequently attributed to activating mutations in the ER gene (*ESR1*) (2–4). Recent in vitro studies suggest that nongenomic mechanisms, including genome-wide reprogramming of the chromatin landscape and DNA methylation changes, contribute to the development of ET resistance (5–8).

Neoadjuvant therapy trials with short-term ET prior to surgery can help to study endocrine resistance. Effective ET inhibits tumor cell growth, as evidenced by a decrease in the Ki67 labeling index (9–11). Persistently high Ki67 expression, despite hormone blockade, indicates estrogen-independent proliferation associated with an elevated risk of disease recurrence and mortality.

The WSG-ADAPT trial, which has enrolled more than 5,600 patients with luminal BC to date, including 2,290 in the ET subtrial, provides a novel approach to investigating endocrine resistance (12). More than 70% of patients in the ADAPT trial responded to short-term ET with either tamoxifen (TAM) or aromatase inhibitors (AI). Nonresponding tumors maintain their original growth rate, making them ideal candidates to study the mechanisms of primary endocrine resistance.

In this study, a discovery cohort and a validation cohort of ET-responsive and nonresponsive patients were subsampled from the WSG-ADAPT trial. We aimed to identify recurrent genomic and epigenomic aberrations associated with primary resistance using targeted NGS and DNA methylation analysis. We hypothesized that these aberrations could provide biomarkers for the aggressive subtype of HR-positive luminal BC and allow the development of predictive models for ET response. We validated our findings in a second validation cohort and the TCGA BRCA cohort and provide survival data for the combined WGS-ADAPT cohorts.

Our results provide insight into the different mechanisms of resistance to TAM and AI and thus suggest a way to overcome resistance by starting with or switching to the drug that is not affected.

## Results

### Study design and patient selection.

**Figure 1:**
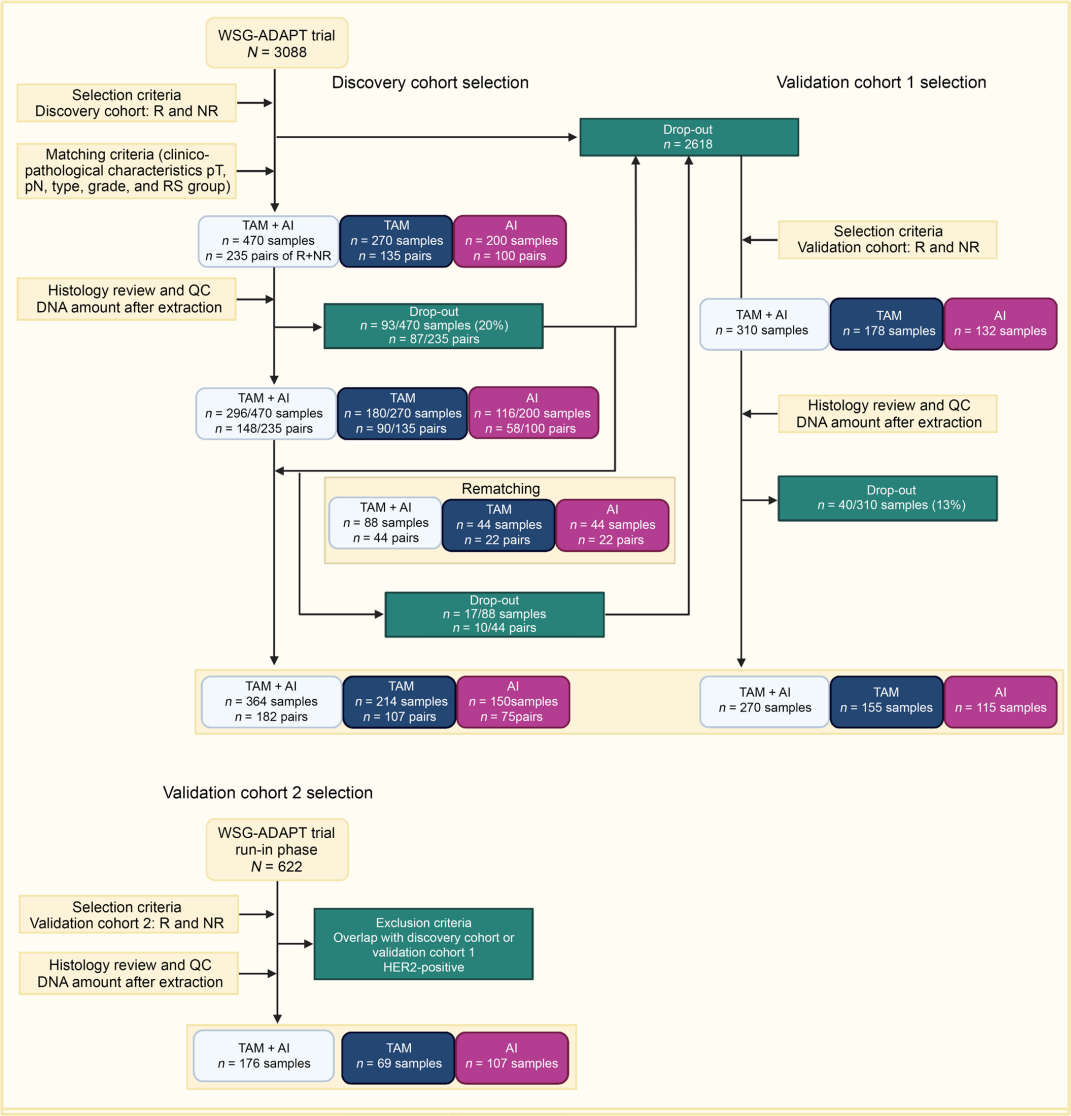
Flowchart of the sample selection. Samples were selected from the WGS-ADAPT trial for the discovery cohort (left, matched sample design), the validation cohort 1 (right, unmatched design), and from the run-in phase of the WGS-ADAPT trial for validation cohort 2 (bottom, unmatched design), respectively.

The WSG-ADAPT trial (NCT01779206) is a phase II, multicenter, controlled, nonblinded, randomly assigned, investigator-initiated trial in the framework of WSG-ADAPT umbrella protocol at the Institute of Pathology, Hannover Medical School (12, 13). We selected early BC patients with ER+ and/or PR+, HER2− tumors, who had received 3 weeks of preoperative ET (pET) (TAM in premenopausal and AI in postmenopausal women). Treatment response was determined by IHC in situ labeling of cycling cells (G1 to M-phase) with Ki67 before and after pET, considering both post-pET Ki67 levels and Ki67 decrease from baseline (Supplemental Table 1; supplemental material available online with this article; https://doi.org/10.1172/JCI177813DS1). In the discovery cohort (*n* = 364, TAM *n* = 214, AI *n* = 150), patients with a post-pET Ki67 less than 10% and a relative decrease greater than or equal to 70% were considered responders (R), and those with a post-pET Ki67 of greater than or equal to 20% and a relative decrease of less than or equal to 20% were considered nonresponders (NR). Response groups were matched for histopathologic features at baseline, including WSG central histologic grade, pT, pN, ER, PR, HER2 status, and oncotype DX recurrence score (RS). We included all possible sample pairs that met our selection criteria (Figure 1 and Supplemental Tables 1 and 2). For validation cohort 1 (*n* = 270, TAM *n* = 155, AI *n* = 115), we relaxed the selection criteria to allow inclusion of additional patients. Responders were defined as a post-pET Ki67 less than 10%, and nonresponders had a post-pET Ki67 greater than or equal to 20%. The response groups were not matched with respect to histopathologic features, RS, or histologic grade (Supplemental Tables 1 and 3). Validation cohort 2 (*n* = 176, TAM *n* = 69, AI *n* = 107) was selected from the run-in phase of the WGS-ADAPT trial (14), using the same criteria as for validation cohort 1 (Supplemental Tables 1 and 4). Inclusion was limited by the amount and quality of available DNA.

### Histologic grade changes correlate with Ki67 staining in response to pET.

**Figure 2:**
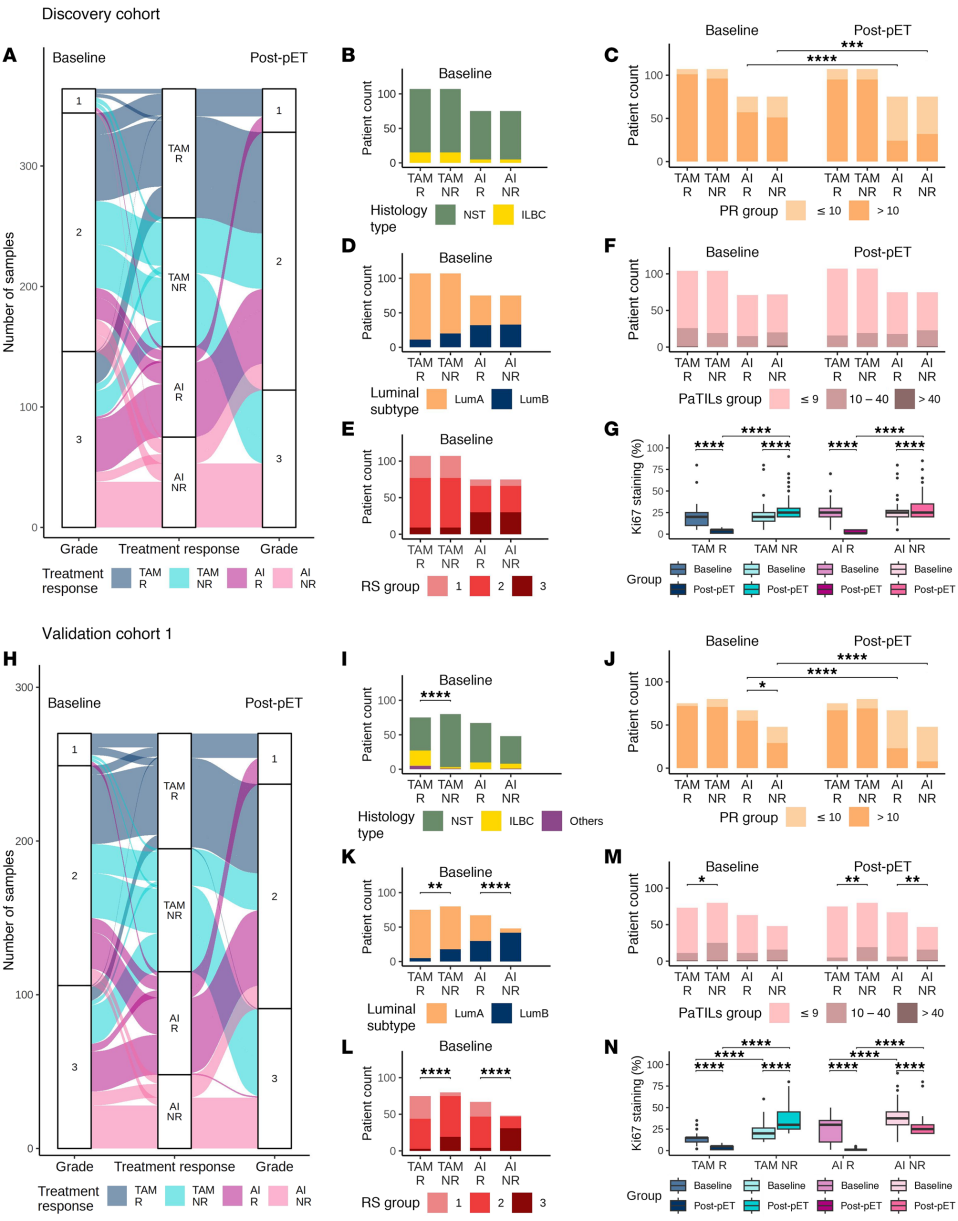
Descriptive statistics of the discovery cohort and validation cohort 1. Distribution of patients (R, responders; NR, nonresponders) according to clinico-pathological parameters before (baseline) and/or after antihormone treatment (post-pET) in the discovery cohort (*n* = 364, TAM *n* = 214, AI *n* = 150) (**A**–**G**) and validation cohort 1 (*n* = 270, TAM *n* = 155, AI *n* = 115) (**H**–**N**). (**A** and **H**) tumor grade; (**B** and **I**) histology type; (**C** and **J**) progesterone receptor (PR) status; (**D** and **K**) luminal subtype; (**E** and **L**) recurrence score (RS); (**F** and **M**) stromal tumor-infiltrating lymphocytes in pathologic tissue sections (PaTILs, patients without PaTIL data were excluded); (**G** and **N**) percentage of Ki67-positive staining in IHC. Statistical differences of numerical variables between matched pairs as well as between baseline and post-pET comparisons were tested using paired Wilcoxon tests. All other comparisons of numerical variables were analyzed using nonpaired Wilcoxon tests. Statistical differences of categorical variables between matched pairs as well as between baseline and post-pET comparisons were analyzed using McNemar test. All other comparisons of categorical variables were analyzed using Fisher’s exact test. For all statistical tests, asterisks *P < 0.05, **P < 0.01, ***P < 0.001, ****P < 0.0001. Boxplots show median (line), upper, and lower quartiles (boxes), and lines extending to 1.5-interquartile range (IQR) (whiskers).

In the discovery cohort (Supplemental Table 2), response to short-term pET was reflected by changes in histologic grade from baseline to post-pET (Figure 2A). Grade decreased in greater than 40% of responders, while many nonresponders increased in grade post-pET (Supplemental Figure 1A). Invasive lobular BC (ILBC) cases were enriched in the TAM group compared with the AI group (Figure 2B). AI treatment resulted in significantly reduced PR staining after pET compared with baseline (Figure 2C). Combining Ki67 and PR staining information, we divided the patients into luminal A and B subtypes. Less than 20% of TAM cases, but more than 40% of AI cases were classified as LumB (Figure 2D). Similarly, TAM-treated patients were predominantly categorized as risk groups RS1 and RS2, whereas AI-treated cases were mainly grouped into RS2 and RS3 (Figure 2E). RS grouping was negatively correlated with PR staining and positively correlated with luminal subtypes (Supplemental Figure 2A and Supplemental Figure 3A). Pathology-derived estimates of tumor-infiltrating lymphocytes (PaTILS) did not differ between the response groups or between pre- and post-pET (Figure 2F), but we observed highly significant differences in Ki67 staining between R and NR cases at baseline and post-pET (Figure 2G).

Since cases in validation cohort 1 were not matched, we observed significant differences in several clinico-pathological parameters (age, histology type, pT, pN, grade, E-cadherin, Ki67, luminal subtype, RS groups) between the discovery cohort and validation cohort 1 at baseline (Supplemental Table 5). pET responders had consistently lower grades at baseline than nonresponders (TAM *P* = 7.48 × 10^–4^, AI *P* = 1.67 × 10^–3^) (Figure 2H). Distinct changes in histologic grading from baseline to post pET reflected response or resistance to pET (Supplemental Figure 1B). TAM responders, but not AI responders, were enriched in ILBC (R 29%, NR 3%) (Figure 2I). We identified significantly more AI nonresponders than responders with weak PR staining prior to pET, which further decreased after AI treatment (Figure 2J). Nonresponders were annotated more often as LumB than as LumA (Figure 2K) and were categorized into higher RS groups than responders (Figure 2L and Supplemental Table 3). RS grouping was positively correlated with grade at baseline and after pET and showed an inverse correlation with PR staining (Supplemental Figure 2B and Supplemental Figure 3B). Infiltrating lymphocytes (PaTIL groups) did not differ significantly between pre- and post-pET (Supplemental Table 3). However, in TAM-treated cases, higher PaTIL levels at baseline and after pET were indicative of resistance, whereas in the AI group, we observed higher PaTIL levels in NR only after pET (Figure 2M). Finally, in contrast to the discovery cohort, Ki67 staining was higher at baseline in nonresponders than in responders (Figure 2N and Supplemental Table 3).

In summary, TAM cases had lower grade and RS scores than AI cases in both cohorts. PR staining inversely correlated with RS grouping. In cases receiving AI, PR staining was consistently lower after pET than at baseline.

### Recurrent TP53 mutations in luminal BC promote resistance to pET.

**Figure 3:**
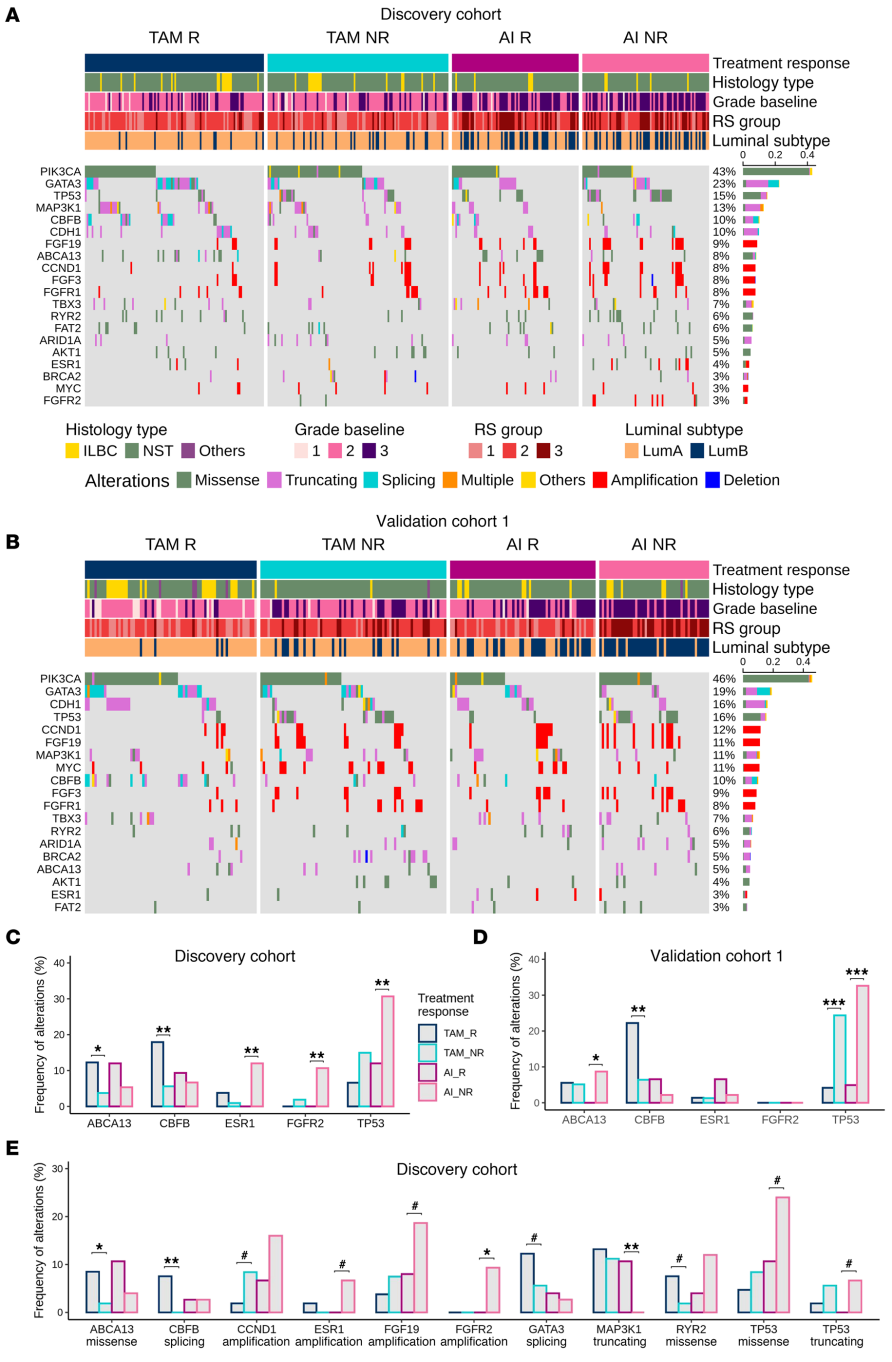
Recurrent genomic alterations. Oncoprints of recurrent genomic alterations (RGA; at minimum 7.5% recurrence in either subgroup) in post-pET samples in the discovery cohort (*n* = 364, TAM *n* = 214, AI *n* = 150) (**A**) and validation cohort 1 (*n* = 270, TAM *n* = 155, AI *n* = 115) (**B**), color-coded by mutation type. Clinical annotations are indicated at the top. Frequencies of RGA recurrence per cohort on the right side. Legend is for both **A** and **B**. (**C** and **D**) Alteration frequencies of selected RGA with significant associations with pET response in the discovery cohort (**C**) and the validation cohort 1 (**D**), analyzed using Fisher-exact test *P < 0.025, **P < 0.01, ***P < 0.001, (**E**) RGA with significant differences between R and NR in the discovery cohort when stratified by alteration type, analyzed using Fisher-exact test with ^#^*P* < 0.1, *P < 0.05, **P < 0.01.

We performed NGS panel sequencing to identify recurrent genomic alterations (RGA) indicating pET resistance (Supplemental Table 6). Mutations of *PIK3CA* and *GATA3*, and amplifications of *CCND1*, *FGF3,* and *FGF19* on chr11q13.3 were most frequently detected in both cohorts (Figure 3, A and B), confirming previous observations (15–18), but not related to pET resistance. *CDH1* is frequently mutated in ILBC (17, 19). We detected *CDH1* frameshift and splice-site mutations in more than 10% of the patients and confirmed significant enrichment of *CDH1* mutations in ILBC cases. *CDH1* mutations were strongly anticorrelated with E-cadherin protein expression (Supplemental Figure 3, A and B).

In the discovery cohort, point mutations in *ABCA13* (12.2% versus 3.7%, *P* = 0.024) and *CBFB* mutations (17.7% versus 5.6%, *P* = 0.006) were more frequent in TAM responders than in nonresponders (Figure 3C). *CBFB* enrichment in TAM R were confirmed in validation cohort 1 (22.2% versus 6.4%, *P* = 0.008) (Figure 3D).

We observed significantly more *ESR1* alterations in AI nonresponders than in AI responders (12% versus 0%, *P* = 0.003), but this observation could not be validated (Figure 3D). *FGFR2* amplifications were also detected more frequently in AI NR (9.3% versus 0%, *P* = 0.014) (Figure 3C) and were positively correlated with grade, Ki67 post-pET and *TP53* mutations (Supplemental Figure 3A). We recently reported a significant association of *TP53* mutations with pET resistance (14, 20). Consistently, significant enrichment of *TP53* mutations in nonresponders in the discovery cohort (TAM *P* = 0.076, AI *P* = 0.009) (Figure 3C) was confirmed in validation cohort 1 (TAM *P* = 0.0004, AI *P* = 0.0002) (Figure 3D).

We analyzed the most prominent RGA separately by alteration type (Figure 3E). We were able to confirm the results of the combined assessment, albeit at a higher significance level for *ESR1* amplifications, *TP53* truncating mutations, and *TP53* missense mutations (*P* < 0.1). Interestingly, when stratifying by mutation type, *MAP3K1* truncating mutations were significantly associated with the AI R group (11% R versus 0% NR, *P* = 0.006). *CCND1* amplifications were more frequent in TAM NR than in TAM R, whereas *GATA3* splicing mutations and *RYR2* missense mutations were more frequent in TAM responders (all at *P* < 0.1).

In conclusion, we found several significant differences in the incidence of RGA between responders and nonresponders. With a frequency of up to 32% in AI NR cases, *TP53* missense and truncating mutations were most commonly associated with primary ET resistance.

### Different alterations in the DNA methylome contribute to resistance to pET with TAM and AI.

Previous studies have reported that ET resistance leads to adaptations in chromatin structure and DNA methylome (5–7). We performed methylation analyses on tumor tissue obtained after pET using EPIC arrays. In the discovery cohort, we detected 472 significantly (*P* < 0.005) differentially methylated CpG sites (DMS) with greater than or equal to 10% mean methylation difference between TAM NR and R groups, and 435 DMS between AI NR and R (Supplemental Figure 4A and Supplemental Table 7). Nearly 70% of the TAM DMS and 40% of the AI DMS were confirmed in validation cohort 1 (Supplemental Figure 4B).

**Figure 4:**
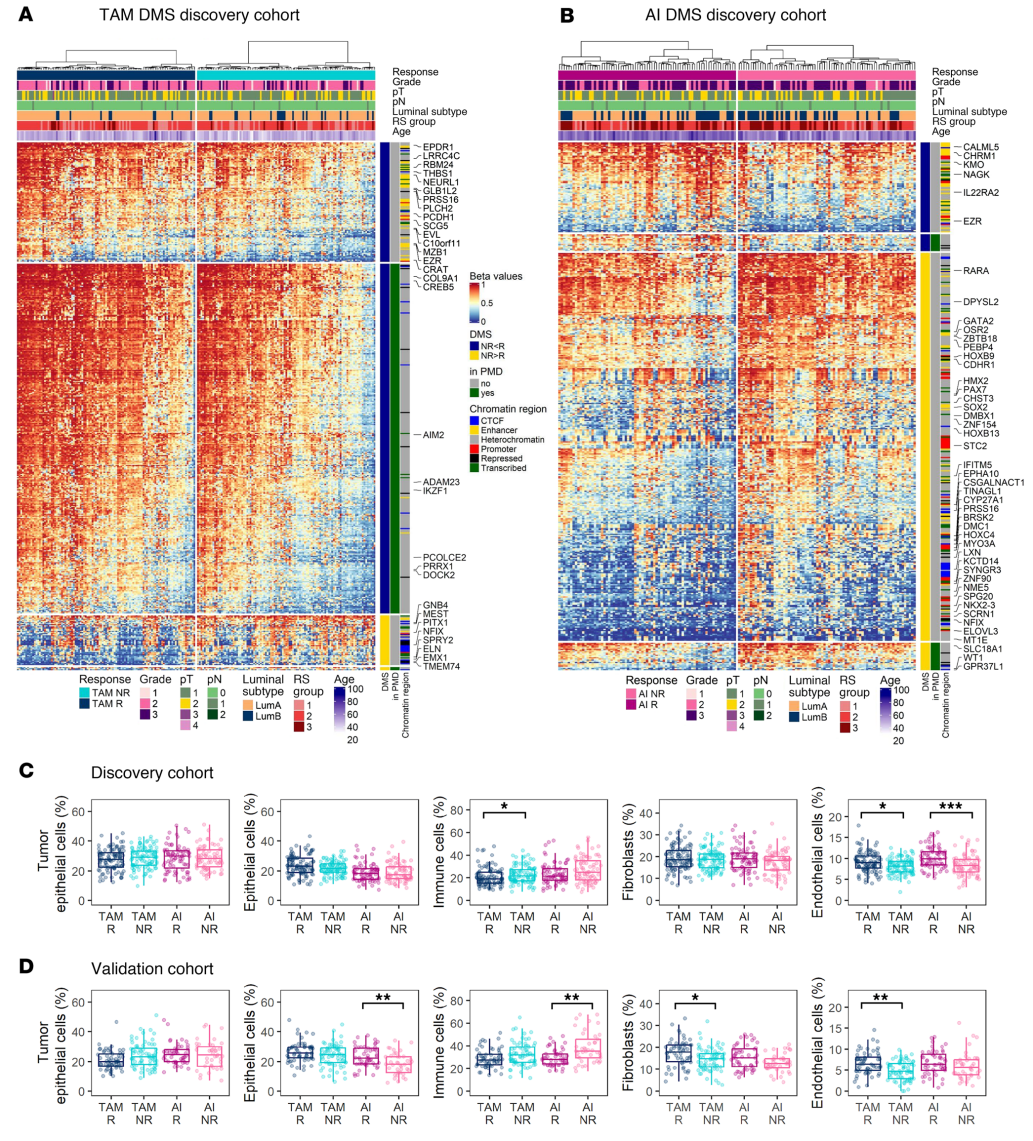
pET-specific alterations in the methylome and tumor microenvironment. (**A** and **B**) Heatmaps of methylation beta values of TAM DMS (**A**) and AI DMS (**B**) in the discovery cohort (*n* = 360, TAM *n* = 210, AI *n* = 150). Clinical annotations are indicated at the top. Gain (NR > R, yellow) and loss (NR < R, blue) in methylation in NR, overlap with PMDs (green) and defined chromatin regions, and gene symbols are indicated on the right side. Rows (CpG sites) and columns (cases) are clustered by Euclidean distance and ward.D linkage. The heatmaps are split by response groups (columns) and by methylation change and location in PMDs (rows). (**C** and **D**) Methylation-derived cell type proportions of BC samples in the discovery (**C**) and validation cohort 1 (**D**). R and NR groups per treatment were compared using Wilcoxon test with fdr-adjusted *P* values: *P < 0.05, **P < 0.01, ***P < 0.001. Boxplots show median (line), upper, and lower quartiles (boxes), and lines extending to 1.5-IQR (whiskers).

We observed distinct patterns of methylation changes in the 2 treatment groups. In TAM-treated cases, 90% DMS were hypomethylated in nonresponders compared with responders (Figure 4A). The majority of TAM DMS (80%) were located in tightly packed heterochromatin and repressed regions (Supplemental Figure 4C, upper panel) that strongly overlapped with partially methylated domains (PMDs) (Figure 4A), megabasepair regions of global methylation loss (21). Annotation of DMS to genes and overrepresentation analysis suggested that TAM pET resistance may be associated with KRAS signaling, apical cell-cell junctions, and epithelial-mesenchymal transition (Supplemental Figure 4D).

In the AI group, approximately 80% DMS were hypermethylated in NR versus R (Figure 4B). Approximately 40% DMS were located in enhancer and promoter regions, suggesting a gene regulatory function, or overlapped with insulator protein CTCF binding or transcribed regions (Supplemental Figure 4C, lower panel). Using data from the TCGA BRCA subcohort to analyze the correlation between methylation and gene expression (Supplemental Table 7), we identified a group of developmental transcription factors with positive correlations, indicating gene upregulation. Additional AI DMS-associated genes were enriched in gene sets related to hypoxia and estrogen response (Supplemental Figure 4D).

We used NanoString BC360 gene expression data to evaluate the effects of methylation on 3 genes linked to DMS (Supplemental Table 8). In the TAM cohort, loss of methylation at cg04334496 in NR was associated with an increase in *EYA4* expression, which has tumor-promoting functions and acts as a transcriptional activator and phosphatase. Conversely, gain of cg16766325 methylation in the promoter region of *SPRY2*, a tumor suppressor with endogenous inhibitory activity for the *RAS*/*MAPK* pathway, was associated with decreased *SPRY2* mRNA expression (Supplemental Figure 4E). cg16766325 methylation negatively correlated with *SPRY2* gene expression also in the TCGA BRCA subcohort (Pearson correlation *r* = –0.423, fdr-adjusted *P* value = 4.11 × 10^–12^, Supplemental Table 7). In the AI cohort, reduced methylation of cg14096855, located in the promoter region of *CALML5*, in the NR group, resulted in a significant upregulation of *CALML5* expression (Supplemental Figure 4F), with stronger inverse correlation in AI nonresponders than in responders (AI-R Pearson′s *r* = –0.309, AI-NR Pearson’s *r* = –0.709, Supplemental Figure 4G).

In summary, we identified distinct methylation changes in the TAM versus AI cohorts. TAM DMS were mostly less methylated in the NR versus R group and were associated with PMDs. Conversely, AI DMS were more methylated in the NR versus R group, located in gene regulatory regions and associated with developmental transcription factors, hypoxia, and estrogen signaling. We identified several BC-related genes with significant inverse correlations between methylation and gene expression.

### pET resistance is associated with changes in composition of the tumor microenvironment.

The tumor microenvironment (TME) may play a role in the development of ET resistance (22). We used a reference-based approach to estimate the TME composition using methylation data. In nonresponders, we consistently saw higher proportions of immune cells and lower proportions of fibroblasts and endothelial cells (Figure 4, C and D), but increased immune cell infiltration was not associated with any particular immune cell type (Supplemental Figure 5, A and B). TIL levels derived from methylation data (MeTILs) correlated significantly with PaTIL levels (Figure 2, E and L, and Supplemental Figure 5C) and with MeTIL scores calculated according to ref. 23 (discovery cohort, ρ=0.638, *P* < 2.2 × 10^–16^; validation cohort ρ=0.697, *P* < 2.2 × 10^–16^).

### Developing the Predictive Endocrine ResistanCe Index.

**Figure 5:**
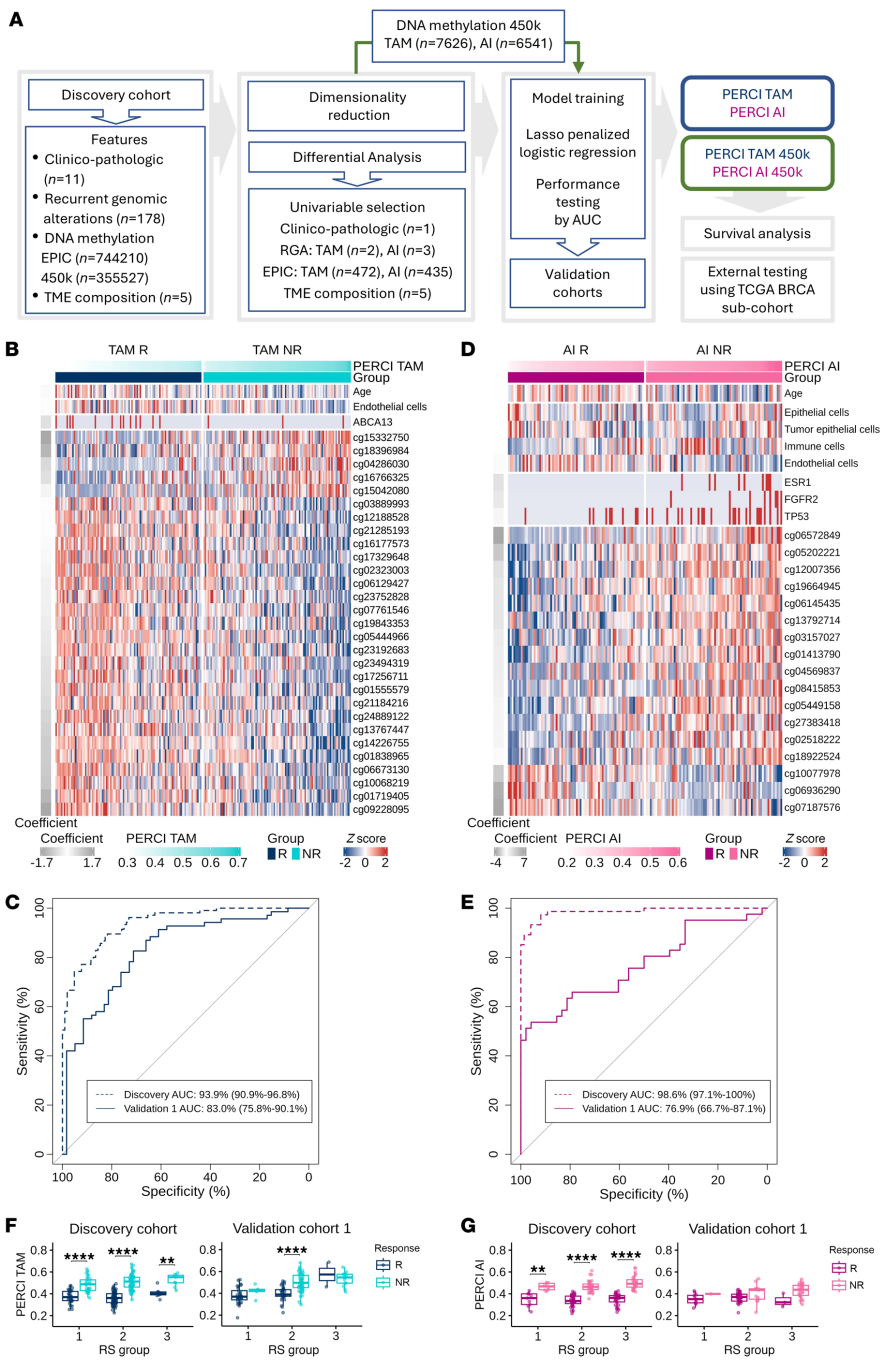
Developing the ‘Predictive Endocrine ResistanCe Index’ PERCI. (**A**) Workflow describing the development of PERCI based on the discovery cohort (*n* = 357, TAM *n* = 209, AI *n* = 148). (**B** and **D**) Heatmap of z-scores for features included in PERCI TAM (**B**) and PERCI AI (**D**). The coefficients on the right indicate the weight of each feature. ROC-AUC analysis of classifier performance for PERCI TAM (**C**) and PERCI AI (**E**) in the discovery cohort (*n* = 357, TAM *n* = 209, AI *n* = 148) and validation cohort 1 (*n* = 217, TAM *n* = 128, AI *n* = 89). The x-axis shows the specificity, while the y-axis shows the sensitivity. ROC-AUC with 95% CI are shown. (**F** and **G**) Performance of PERCI TAM (**F**) and PERCI AI (**G**), stratified by RS subgroups, in the discovery (left) and validation cohort 1 (right). R and NR groups per treatment were compared using (discovery cohort: paired) Wilcoxon test with FDR-adjusted *P* values: **P < 0.01, ***P < 0.001, ****P < 0.0001. Boxplots show median (line), upper, and lower quartiles (boxes), and lines extending to 1.5-IQR (whiskers).

After identifying genomic and epigenomic differences between responders and nonresponders in the discovery cohort, we used lasso penalized logistic regression to train classifiers for TAM and AI resistance, which we named Predictive Endocrine ResistanCe Index (PERCI) (Figure 5A). PERCI TAM consists of age information, endothelial cell content, *ABCA13* mutations, and methylation data for 29 TAM DMS with different weights (Figure 5B and Supplemental Table 1). Most of the DMS were hypomethylated in the nonresponder groups, and about half of them were located in heterochromatic regions (Supplemental Table 1 and Supplemental Table 7). Five PERCI TAM DMS (cg15042080, cg16766325, cg04286030, cg18396984, and cg15332750) were hypermethylated in nonresponders. The individual predictors achieved an area under the receiver operating characteristic curve (ROC-AUC) of 54.8% (*ABCA13* mutations) to 72% (cg01838965) (Supplemental Figure 6). Combining all features, PERCI TAM stratified response groups with a ROC-AUC of 93.9% (Figure 5C), with excellent accuracy and positive predictive value (Table 1). Neither clinico-pathological parameters (except for age) nor RS groups showed significant differences between responders and nonresponders (ROC-AUC ranging from 42.4% to 58.5%, Supplemental Table 9) and were therefore not included in the PERCI model.

Strong performance of PERCI TAM was confirmed in validation cohort 1, with a ROC-AUC of 83%, which outperformed clinico-pathological parameters (ROC-AUC in a range of 44.8% [ER% baseline] to 72.6% [RS group information], Supplemental Table 10). Testing the frequencies of correctly and incorrectly annotated cases in a confusion matrix suggests that PERCI TAM is better at predicting NR than R in both cohorts.

PERCI AI includes patient age, *ESR1*, *FGFR2*, and *TP53* genomic alterations, several cell type proportions, and methylation information for 17 AI DMS, most of which gained methylation in AI nonresponders (Figure 5D). Hypermethylation of cg18922524 in the *HOXC4* promoter was most positively correlated with *HOXC4* mRNA levels indicating increased gene expression (Pearson correlation *r* = 0.475, fdr-corrected *P* value = 1.13 × 10^–15^) among 12 AI DMS associated with the same gene (Supplemental Table 1 and Supplemental Table 7).

The DMS selected for PERCI AI achieved ROC-AUC values from 67.1% to 74.4% (Supplemental Figure 7). In combination, the features stratified the response groups with 98.6% ROC-AUC and excellent accuracy and true positive rate (Figure 5E and Table 1). Like PERCI TAM, PERCI AI was better at predicting NR than R.

In validation cohort 1, we obtained a ROC-AUC of 76.9%, and PERCI AI was better at correctly predicting the responder group. Several clinico-pathological parameters with significant differences between responders and nonresponders in validation cohort 1 (PR% baseline, Ki67, RS group, Luminal subtype, Figure 2) also stratified the response groups with AUC-ROC above 70%. For RS group information, we calculated an AUC-ROC of 83.6% (Supplemental Table 10).

In the discovery cohort, both PERCI TAM and PERCI AI effectively discriminated between R and NR, even when the cohort was split into RS groups, despite RS being used in the matched-pair design (Figure 5, F and G). We could validate PERCI TAM results in RS2 in validation cohort 1 (Figure 5F). PERCI TAM and PERCI AI both correlated strongly with post-pET histology grade and Ki67 staining (Supplemental Figures 2, A and B).

In summary, our novel predictors PERCI TAM and PERCI AI combine information on genomic alterations, patient age, TME composition, and differential methylation, with equal or better performance than existing predictors such as the Oncotype DX recurrence score. ROC-AUCs of 83% for TAM and 76.9% for AI confirmed the predictive performance of PERCI in validation cohort 1.

### Adapting PERCI to the Illumina 450k platform and validation in a second WGS-ADAPT trial validation cohort.

The multi-criteria nature of PERCI may limit its applicability in clinical settings where not all data modalities are readily available. Therefore, we trained a streamlined version of PERCI, using only methylation data and age. We restricted CpG sites to those that overlapped between EPIC and the widely available Illumina 450k array, with mean methylation differences of greater than or equal to 5% and various significance thresholds (Methods). PERCI TAM 450k contains age and methylation information for 40 CpG sites mostly hypomethylated in TAM-NR (Supplemental Figure 8A and Supplemental Table 1). Ten CpGs overlapped with PERCI TAM features. For PERCI AI 450k, in addition to age, 19 predominantly hypermethylated CpG sites were selected, 8 of which overlapped with PERCI AI features (Supplemental Figure 8B and Supplemental Table 1). Both 450k-compatible versions of PERCI performed as well as or better than PERCI in the ADAPT trial subcohorts, with ROC-AUC of greater than 95% in the discovery cohort and 80.5% (TAM) and 85.5% (AI) in validation cohort 1 (Table 2 and Supplemental Figure 8, C and D). When we stratified the cohorts by RS groups, both simplified indices performed very well (*P* < 0.01) in discriminating the response groups across all RS groups in the discovery cohort, and in RS2 in validation cohort 1 (Supplemental Figure 8, E and F).

**Figure 6:**
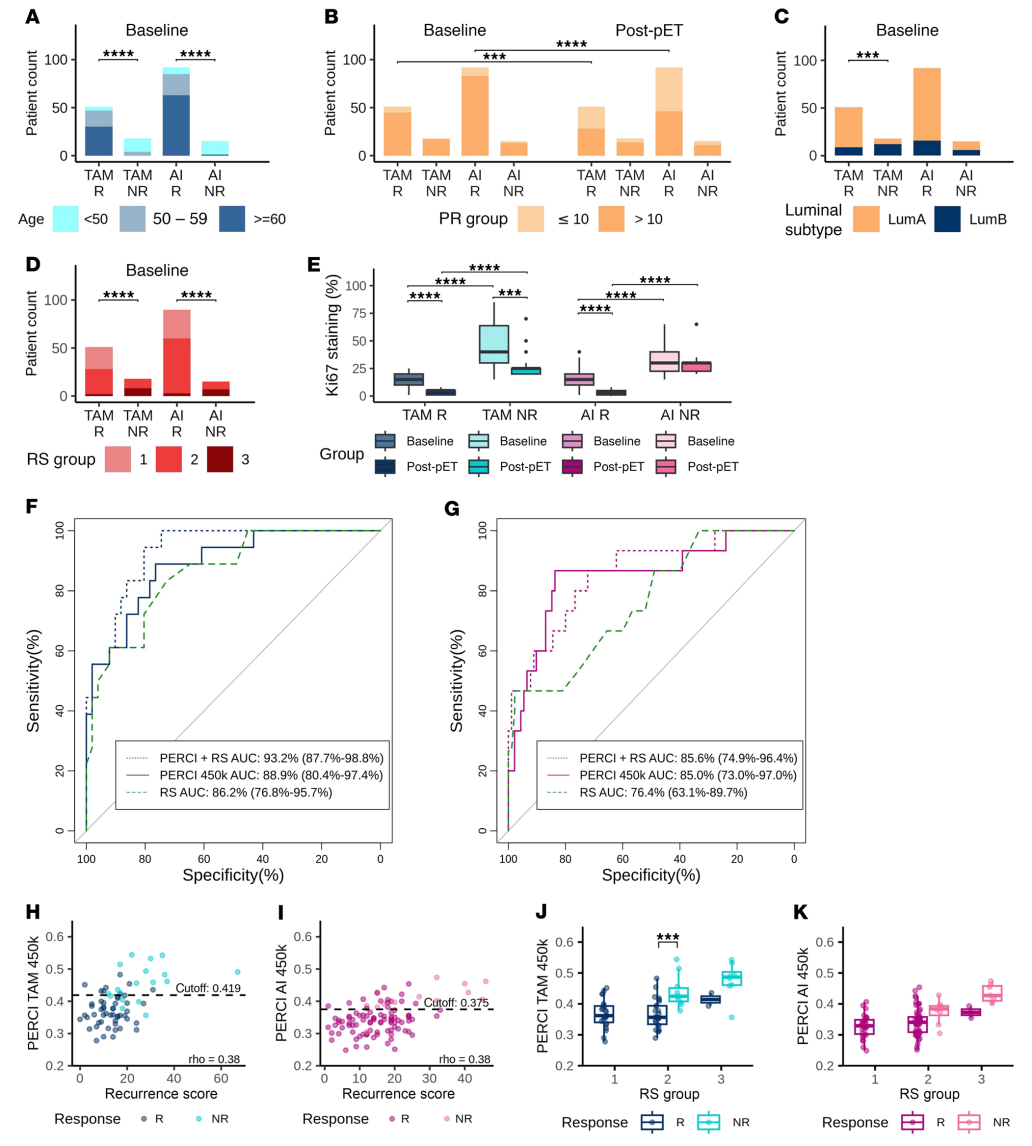
Descriptive statistics and performance of PERCI TAM 450k and PERCI AI 450k in validation cohort 2. Clinico-pathological parameters with significant differences between response groups at baseline and/or post-pET in validation cohort 2 (*n* = 176, TAM *n* = 69, AI *n* = 107). (**A**) age; (**B**) PR status; (**C**) luminal subtype; (**D**) RS groups; and (**E**) Ki67 staining. Statistical tests as described in the legend of Figure 2. Statistical differences of numerical variables between baseline and post-pET comparisons were tested using paired Wilcoxon tests. All other comparisons of numerical variables were analyzed using nonpaired Wilcoxon tests. Statistical differences of categorical variables between baseline and post-pET comparisons were analyzed using McNemar test. All other comparisons of categorical variables were analyzed using Fisher’s exact test. For all statistical tests, asterisks ***P < 0.001, ****P < 0.0001. (**F** and **G**) Analysis of the performance of PERCI 450k TAM and RS, alone and in combination (**F**) and PERCI 450k AI and RS, alone and in combination (**G**) by ROC-AUC (PERCI 450k: black solid line; RS, black stippled line; combination of PERCI 450k and RS, green dashed line). The x-axis shows specificity and the y-axis shows sensitivity. ROC-AUC with 95% CI are given. (**H** and **I**) Scatter plot of Recurrence Score versus PERCI TAM 450k (**H**) and PERCI AI 450k (**I**). The vertical black lines indicate treatment-specific cutoffs for PERCI 450k to discriminate between responders and nonresponders. Spearman correlation coefficient ρ as indicated. (**J** and **K**) Performance of PERCI TAM 450k (**J**) and PERCI AI 450k (**K**) in validation cohort 2, stratified by RS groups. R and NR groups per treatment were compared using Wilcoxon test with FDR-adjusted *P* value: ***P < 0.001. Boxplots show median (line), upper, and lower quartiles (boxes), and lines extending to 1.5-IQR (whiskers).

To test PERCI 450k in an independent validation cohort, we selected patients from the WGS-ADAPT run-in period (Supplemental Tables 1 and 4). Validation cohort 2 included more responders (81%) than nonresponders (19%). We observed significant age differences between response groups, with nonresponders being predominantly under 50 years of age (Figure 6A and Supplemental Table 4). Both responder groups showed reduced PR staining compared to baseline (Figure 6B). TAM nonresponders were highly enriched (67%) for the LumB subtype. In validation cohort 2, we detected less LumB cases in the AI-treated groups (R: 17%, NR: 40%) than in the discovery cohort (R: 43%, NR: 44%) and validation cohort 1 (R: 44%, NR: 87%) (Figure 6C and Supplemental Tables 1–4). RS groups and Ki67 staining levels at baseline were significantly higher in nonresponders than in responders (Figure 6, D and E). Consequently, age and Ki67 at baseline stratified the response groups with very high ROC-AUC ranging from 87.9% to 94.9% (Supplemental Table 11).

Both simplified PERCI 450k scores demonstrated very good performance in stratifying the response groups, with ROC-AUC values of 88.9% for PERCI TAM 450k and 85% for PERCI AI 450k (Figure 6, F and G). Recurrence Scores also performed very well in validation cohort 2 (Figure 6, F and G). Notably, combining PERCI 450k and recurrence scores increased the discriminatory potential from ROC-AUC 88.9% to 93.2% in the TAM cohort. In the AI cohort, the combined analysis did not substantially improve the results beyond PERCI AI 450k alone (ROC-AUC 85% versus 85.6%). Direct comparisons of PERCI 450k and recurrence scores revealed positive correlations between the 2 indices. For both the TAM- and AI-treated groups, the Spearman’s ρ was 0.38 (Figure 6, H and I). Assigning patients to RS groups revealed significant differences (*P* < 0.001) in PERCI TAM 450k in RS2 (Figure 6J), confirming the result from validation cohort 1 (Figure 5F). We observed a trend of higher mean PERCI AI 450k values in nonresponders of RS2 and RS3 in AI validation cohort 2, although the results were not statistically significant (Figure 6K).

These data demonstrate the robustness of our newly developed classifiers and suggest that they could improve the effectiveness of existing patient stratification tools such as the Recurrence Score.

### External validation of PERCI 450k in the TCGA BRCA cohort.

For further testing in an external cohort, we selected 269 cases from the TCGA BRCA cohort that met our selection criteria (Methods). Patients were stratified by menopausal status into a TAM-like cohort (*n* = 75) and an AI-like cohort (*n* = 194) (Supplemental Tables 12 and 13). Consistent with NGS results for the WSG-ADAPT subcohorts, these cases have recurrent mutations of *PIK3CA, TP53, CDH1*, *RYR2, GATA2,* and *FGFR1* and amplification of chr11q13.3 (Supplemental Figure 9). Mutations in *CBFB*, which represented a marker of good response in the TAM cohorts, were less frequent in the TCGA subcohort (3% versus 10% in the discovery cohort and validation cohort 1).

**Figure 7:**
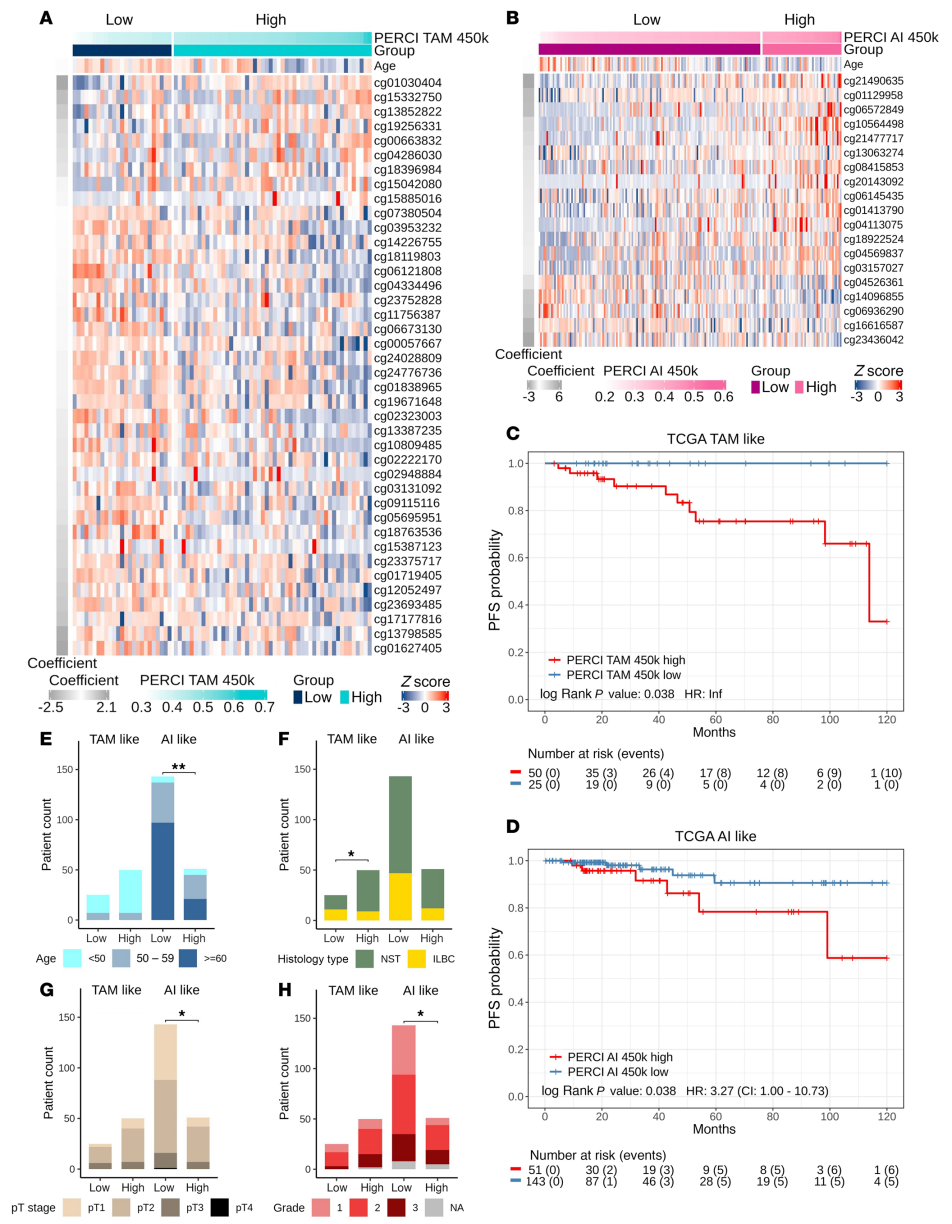
PERCI 450k predicts progression-free survival (PFS) in the TCGA BRCA subcohort. Heatmap of PERCI TAM 450k (**A**) and PERCI AI 450k (**B**) features in the TCGA BRCA subcohort (*n* = 269, TAM-like *n* = 75, AI-like *n* = 194). Kaplan-Meier curves of PFS in the TCGA BRCA subcohort on the basis of PERCI TAM 450k (**C**) and PERCI AI 450k scores (**D**). Cases were divided into high and low groups (blue: low, good prognosis, red: high, poor prognosis) by the cut-off value 0.423 for PERCI TAM 450k and 0.4011 for PERCI AI 450k. *P* values were calculated using the log-rank test. (**E**–**H**) Comparative clinical pathology of PERCI 450k low and high groups in the TCGA BRCA subcohort by (**E**) age; (**F**) histology type; (**G**) pathological stage; and (**H**) tumor grade. Statistical differences of variables were analyzed using Fisher’s exact test *P < 0.05, **P < 0.01.

We divided the TCGA BRCA subcohort into PERCI 450k high and low groups, using progression-free survival within 10 years as a surrogate for ET response (PERCI TAM 450k high *n* = 50, low *n* = 25, Figure 7A). A Kaplan-Meier plot showed excellent stratification of these subgroups. Patients in the PERCI TAM 450k low group had a favorable prognosis with no disease progression within 10 years (log-rank *P* = 0.03, Figure 7C). In addition, there were significantly more ILBC cases in the PERCI TAM 450k low group than in the PERCI TAM 450k high group (*P* = 0.032, Figure 7F), confirming our observations in validation cohort 1 (Figure 2I). Clinico-pathological parameters classified the prognosis groups with ROC-AUC in the range of 42.6% to 73.6% (Supplemental Table 14).

PERCI AI 450k high (*n* = 51, Figure 7B) had a significantly worse prognosis than the low group (*n* = 143), with a hazard ratio of 3.27 for disease progression (log-rank *P* = 0.038, Figure 7D). Cases in the PERCI AI 450k high group were younger (*P* = 1.9 × 10^–3^) and had higher stage (*P* = 0.026) and histologic grades (*P* = 0.026) compared with the PERCI AI 450k low group (Figure 7, E, G, and H, and Supplemental Tables 12 and 13). ROC-AUC for clinico-pathological parameters were in the range of 44% to 65.2% (Supplemental Table 14).

In conclusion, these results suggest that PERCI may not only be a predictor of primary endocrine resistance but may also have prognostic value.

### PERCI predicts survival outcomes in the combined discovery and validation cohorts.

The recent availability of survival data in the WSG-ADAPT trial allowed us to investigate the prognostic value of PERCI. For PERCI, patients from the discovery and validation cohort 1 were combined; for PERCI 450k we also added patients from validation cohort 2. The median follow-up duration was 59.8 months, so the number of invasive and distant disease-free survival (IDFS, DDFS) events and deaths is still limited (combined TAM cohorts: 30 [8.2%], 27 [7.4%], 13 [3.5%]; combined AI cohorts: 35 [11.8%], 32 [10.7%], 17 [5.7%], Table 3). Univariate Cox proportional hazards models indicated that patients with high PERCI TAM or PERCI TAM 450k had a significantly increased risk for disease progression or death, whereas the PERCI AI scores predicted a significantly increased risk for IDFS, and the simplified PERCI AI 450k additionally for death (Table 3).

**Figure 8:**
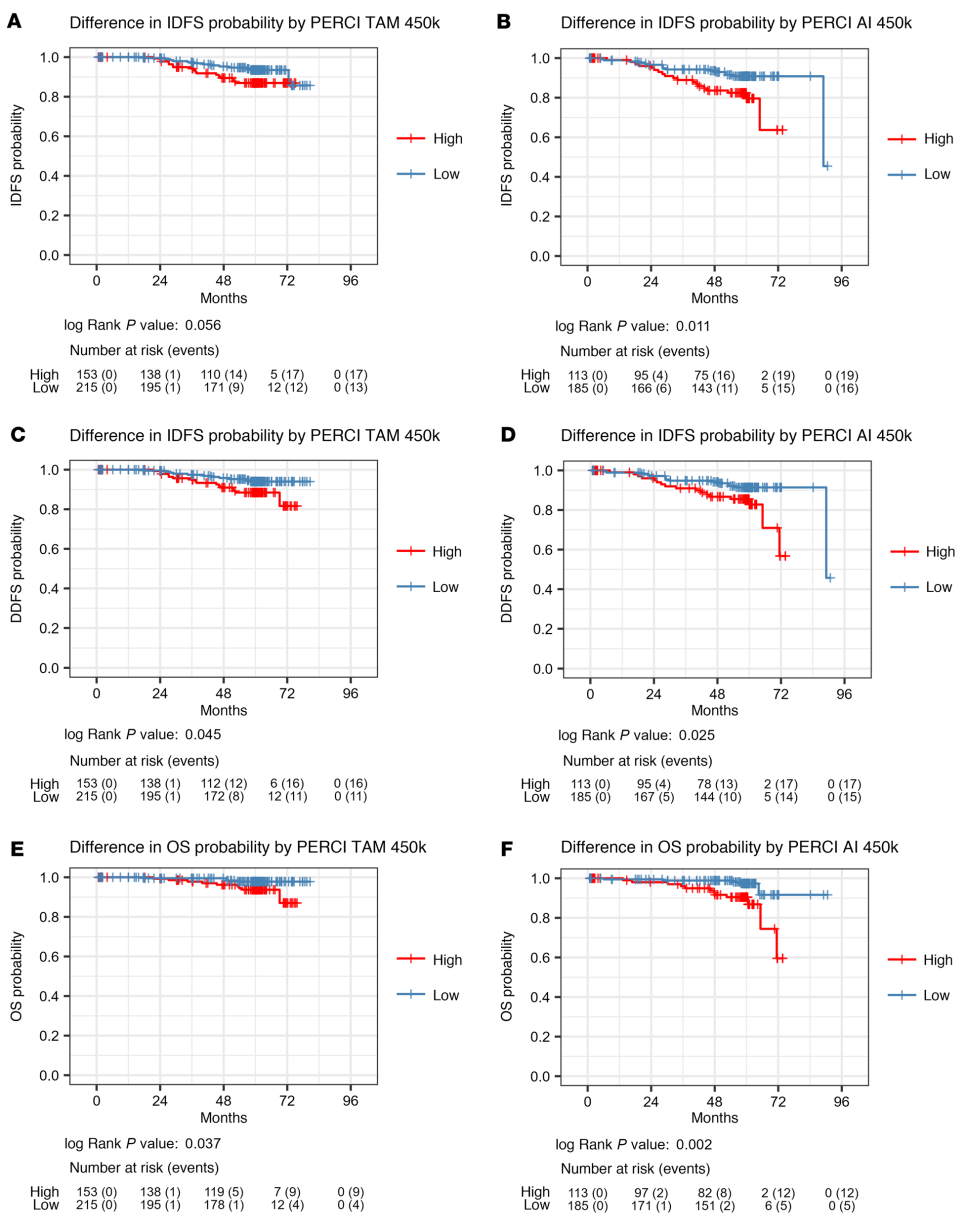
PERCI 450k predicts IDFS, DDFS and OS in the combined discovery and validation cohorts. (**A** and **B**) Kaplan-Meier curves of invasive disease-free survival (IDFS), (**C** and **D**) distant disease-free survival (DDFS) and (**E** and **F**) overall survival (OS) on the basis of PERCI TAM 450k (**A**, **C**, and **E**) and PERCI AI 450k (**B**, **D**, and **F**). Cases were divided into high and low groups using the treatment-specific cutoffs indicated below the Kaplan-Meier curves (blue: low PERCI 450k, good prognosis, red: high PERCI 450k, poor prognosis). *P* values were calculated using the log-rank test. Cases with clinical follow-up: *n* = 666 (TAM *n* = 368, AI *n* = 298).

We divided the patients into high and low PERCI 450k groups according to treatment-specific cutoffs (TAM: high group 41.6% of patients, low group 58.4% of patients; AI: high group 37.9% of patients, low group 62.1% of patients). Kaplan Meier analyses showed that for all survival outcomes, the risk of an event was significantly higher in the high groups than in the low groups (TAM: IDFS log rank *P* = 0.056, DDFS log rank *P* = 0.045; OS log rank *P* = 0.037; AI: IDFS log rank *P* = 0.011, DDFS log rank *P* = 0.025; OS log rank *P* = 0.002) (Figure 8).

## Discussion

The clinical definition of endocrine resistance in BC implies progression or relapse during ET over a period of 6 months to 2 years (24). If a test for primary endocrine resistance were available prior to ET initiation, inadequate therapy could be avoided or substituted. Therefore, this study aimed to characterize and compare molecular alterations associated with primary resistance to TAM and AI treatment in order to develop classifiers for predicting treatment response and survival.

Primary endocrine resistance can be assessed by a diminished or absent proliferative BC response to short-term pET, as evidenced by in situ detection of the proliferation-associated nuclear Ki67 antigen (9, 12, 25). Luminal BC is highly heterogeneous, requiring analysis of many cases. Our study included 810 patients in 3 cohorts and had greater power to detect significant differences between response groups than previous smaller studies (26, 27). We believe that our study design was exceptional. It used tumor tissue from the unique WGS-ADAPT prospective clinical trial and applied different selection criteria to the discovery and 2 validation cohorts. In particular, the discovery cohort included an equal number of pET responders and nonresponders, in contrast with previous studies (16, 26), and the response groups were precisely matched for clinico-pathological parameters to exclude confounding effects related to differences in baseline tumor characteristics. For example, both response groups had similar proportions of G3-differentiated BCs (Figure 2A and Supplemental Table 2). Thus, subsequent molecular analyses were informative of pET response determinants and were not biased by dominant molecular features associated with G3 differentiation. In addition, the criteria for endocrine response and resistance were particularly stringent. In contrast, the validation cohorts had more relaxed selection criteria and were more representative of the patient populations encountered in clinical practice. Therefore, our combined study design was well suited to identify informative markers of preoperative treatment failure in the discovery cohort and to validate them under representative clinical conditions in the validation cohorts.

The mechanisms underlying primary ET resistance can be diverse (28). To develop PERCI, we combined clinico-pathological information with data on significant RGA and pET resistance-specific epigenetic alterations. To select the most informative features, we used lasso penalized logistic regression (29) (Figure 5A). For PERCI AI, the algorithm selected genomic alterations in *ERS1,* *FGFR2,* and *TP53*, key cancer driver genes that were enriched in AI nonresponders, while PERCI TAM was based on a higher mutation frequency of *ABCA13* in TAM responders.

Somatic mutations associated with primary resistance are often detected by their prevalence in metastatic lesions when compared with primary BC (30). Significant progress has been made with the discovery of *ESR1* mutations, which are detectable in less than 1% of primary BC, but are enriched in metastatic luminal BC during adjuvant ET (in up to 15%–30%) and lead to ligand-independent autocrine tumor cell growth. In our discovery cohort, we detected *ESR1* mutations and amplifications in 12% of AI nonresponders, but in none of the AI responders (in total 4%). Consistently, Ferrando et al. detected *ESR1* amplifications enriched in metastatic lesions of BC cases compared with primary tumors exclusively in patients treated with adjuvant AI, but not in TAM-treated patients (31). Gene expression analysis in a subset of our discovery cohort indicated that the increased expression of *ESR1* mRNA in cases with *ERS1* amplification was already detectable in treatment-naive cases at baseline prior to pET and is therefore not treatment related (not shown). The observed accumulation of *ESR1* RGA in AI nonresponders may therefore be a chance finding that was not reproduced in validation cohort 1.

We found that *FGFR2* amplifications were enriched in AI nonresponders. Mao et al. previously reported an increase in *FGFR2* amplification in postresistance biopsies treated with ER-targeted therapy (32). Similarly, *TP53* mutations were detected in greater than 25% of metastases from luminal BC (30). Our method of identifying primary BC resistance by assessing lack of proliferative response to short-term ET identified *TP53* as the most frequently mutated gene associated with treatment failure in the discovery cohort (Figure 3C). These findings were confirmed for both treatment regimens in validation cohort 1 (Figure 3D). These results suggest that the 2 approaches for assessing mechanisms of endocrine resistance provide overlapping information and support the relevance of these RGA as features in PERCI. Consistently, Gellert et al. found reduced suppression of *Ki67* in poor responders with *TP53*-mutant ER-positive BC treated with AI for 2 weeks (26). Since *TP53* mutations lead to aberrant nuclear accumulation of the mutant p53 protein (33), p53 IHC may potentially be used as a surrogate marker of endocrine resistance in a clinical setting (20).

*ABCA13* mutations were included as a feature in PERCI TAM and indicated a better response to treatment (Figure 3C). ABC transporters contribute to therapy resistance through ATP-dependent drug efflux (34). Therefore, inactivation by point mutations could potentially improve response to therapy. Gellert et al. reported a slightly increased (but not statistically significant) prevalence of *ABCA13* mutations in patients responding to AI treatment (26).

In addition to genomic alterations and age, PERCI includes DNA methylation differences between responders and nonresponders and cell type composition (Figures 4 and 5). CpG sites selected for PERCI TAM were predominantly hypomethylated in TAM nonresponders and located in PMDs. Global methylation loss is associated with accelerated cell proliferation and the inability of a cell to remethylate DNA in late-replicating regions after DNA doubling. This leads to the formation of PMDs in the nuclear periphery (21). cg04334496, located downstream of *EYA4,* is an example of methylation loss linked to gene upregulation (Supplemental Figure 4E). In a recent report, over-expression of *EYA4* in breast tissue resulted in an aggressive and invasive BC phenotype (35). In contrast, cg16766325 in the *SPRY2* promoter was hypermethylated in TAM nonresponders, indicating *SPRY2* downregulation (Supplemental Figure 4E and Supplemental Table 7). *SPRY2* inhibits cell proliferation by acting as a feedback inhibitor of the RAS-MAPK pathway downstream of FGF/FGFR (36), and loss of *SPRY2* expression was shown to promote cancer-associated fibroblast activation and BC progression (37). Consistent with these findings, KRAS signaling, apical cell-cell junctions, and epithelial-mesenchymal transition were identified as the most enriched gene sets associated with differential methylation in the TAM group (Supplemental Figure 4D) and may promote TAM resistance (28, 38, 39).

The DNA methylome associated with AI resistance was characterized by a predominant gain in methylation. For a subset of the AI DMS, methylation correlated positively with gene expression. Many of these hypermethylated, upregulated genes belong to the developmental transcription factor family (40) and have previously been implicated in BC etiology and endocrine resistance (41), including *GATA2* (42), *HOXC4* (43), *HOXB13* (44), *HOXC13* (45), *MNX1* (46), *OTX1* (47), *PAX7*, *SOX2* (48), and *WT1* (49). Notably, 1 of the 12 AI DMSs associated with *HOXC4* was included in both AI models. Consistent with our findings, *HOXC4* hypermethylation was identified as a biomarker of endocrine resistance in a small study of 31 ET-treated TCGA BRCA cases (50). The authors also associated *EPSTI1* promoter hypermethylation with endocrine resistance, and 2 AI DMSs (cg01536987, cg22905097) overlapped with this region. *EPSTI1* is overexpressed in aggressive BC and may confer breast stem/progenitor cell properties (51). In our study, methylation of cg01536987 was inversely correlated with *EPSTI1* expression (Supplemental Table 7). cg14096855 was selected as 1 feature in PERCI AI 450k. Methylation of this CpG was particularly low in cases with the highest PERCI AI 450k scores (Figure 7B), and low methylation in a subset of AI nonresponders was associated with strong upregulation of *CALML5* (Supplemental Figure 4, F and G). Single-cell expression analysis in BC recently identified *CALML5* as a marker of epithelial-mesenchymal plasticity and related to metastatic potential (52). Another study associated high *CALML5* expression with treatment failure in a study on HR+/HER2+ BC (53). Functionally, the association between high CALML5 expression and AI resistance needs to be further investigated.

AI DMS-associated genes were enriched in gene sets related to hypoxia (54) and estrogen response (Supplemental Figure 4D). These findings suggest that resistance to AI therapy may be due to an altered response to hypoxic conditions. In a previous report by Oshi et al., 3 months of neoadjuvant AI was shown to reduce the expression of *HIF-1*, a master regulator of oxygen homeostasis (55). Oshi et al. also linked low expression of early estrogen response genes to a reduced ET response (55). Consistently, reexpression of the epigenetically silenced early estrogen response gene *ELOVL2* rescued its downstream signaling and TAM sensitivity in TAM-resistant MCF7 cells and in a xenograft mouse model (56). In our study, cg14153064 located in the *ELOV2* promoter was significantly hypermethylated in AI nonresponders (Supplemental Table 7).

Previous research on epigenetic mechanisms in ET resistance have focused on cell culture models of acquired resistance to ER antagonists. Similar to our observations with TAM, early work by Fan et al. reported predominant hypomethylation rather than methylation gain in long-term cultured HR+ BC cell lines (5). Magnani et al. identified a switch from ER to NOTCH-PBX1 signaling and reprogramming of the chromatin landscape in ET-resistant cell lines and developed a 25-gene signature including the homeobox transcription factor *PBX1* to predict patient outcome (6). Interestingly, our list of TAM DMS includes cg17274057, which is located in an enhancer region of *PBX1* and is hypomethylated in TAM nonresponders.

Stone et al. postulated that hypermethylation of ER-responsive enhancers defines endocrine sensitivity in BC (7). In contrast with these data, we identified hypomethylation associated with TAM resistance mainly in nonenhancer regions, and, conversely, AI DMS located in enhancer regions were hypermethylated predominantly in nonresponders, suggesting that mechanisms of acquired resistance may differ from those of intrinsic ET resistance.

DNA methylation is a stable epigenetic mark of cellular identity (57). We used reference DNA methylomes to infer TME composition. Proportions of bioinformatically estimated MeTILs highly correlated with PaTIL levels in 2 cohorts (Supplemental Figure 5). Proportions of normal and tumor epithelial cells, immune cells, and endothelial cells were selected as features of PERCI AI, albeit with minor contributions to the model. We also noticed that samples with *TP53* mutations were enriched in immune cells, suggesting an interaction between features.

Limitations to the use of PERCI in clinical settings may include sample size, quality of DNA from FFPE tissue, or instrumentation and budget constraints that limit the generation of high-quality NGS data (58). To circumvent these limitations, we generated a simplified version of PERCI, PERCI 450k, including only DNA methylation features and patient age (Supplemental Figure 8). The feasibility of using DNA methylation as a cost-effective classifier in clinical samples has been demonstrated in brain tumors (59), where methylation-based profiling is now part of the WHO classification of central nervous system tumors (60). In our cohorts, PERCI 450k performed as well as or better than PERCI in stratifying responders and nonresponders, with ROC-AUCs above 80% in the validation cohorts (Figure 6 and Table 2). In addition, PERCI 450k showed promise as a prognostic marker in predicting progression-free survival in the TCGA BRCA subcohort (Figure 7) and in our combined cohorts (Figure 8).

The Oncotype DX Recurrence Score (RS) is a test that estimates the risk of distant recurrence based on the expression of 21 genes (61). It is now widely used in the clinics to help guide treatment decisions. We could demonstrate that PERCI 450k and Recurrence Scores are positively correlated. Our results suggest that combining both indices may improve the accuracy of luminal BC patient stratification, especially in TAM-treated cases, where PERCI or PERCI 450k consistently discriminated response groups, particularly in the intermediate-risk group RS2.

### Conclusions.

This study provides evidence that the cellular pathways of endocrine resistance to TAM and AI are influenced by distinct genetic and epigenetic alterations. The delineation of differences between the mechanisms of primary endocrine resistance between the 2 mainstays of standard ET in BC opens the perspective of overcoming resistance by starting with the drug not affected by resistance markers or subsequently switching from one drug to the other. PERCI, as a validated biomarker of endocrine resistance, can be readily used for risk stratification in future therapeutic trials in BC.

## Methods

### Sex as a biological variable.

BC in men is rare, with only 0.5%–1% of BC cases occurring in men (62). Therefore, this study focuses on BC in women.

Participants in the WGS-ADAPT trial are predominantly white. Race was not considered as a biological variable.

### Clinico-patholocial data acquisition.

FFPE specimens were prepared from baseline and post-pET tumor biopsies from all patients and were submitted for histologic rereview and immunohistochemistry to the ADAPT Study Central Reference pathology at Hannover Medical School. Expression of ER, PR, HER2, and Ki67 protein were assessed using standardized methods (12). Tumors with baseline Ki67 ≥ 35% or PR ≤ 20% were classified as LumB subtype, all other tumors were designated as LumA. RNA was extracted from baseline samples and the Oncotype DX RS was determined at the Genomic Health Inc. laboratory. Nanostring Breast Cancer 360 mRNA expression was analyzed on a nCounter FLEX system (Nanostring technology) for a subset of the discovery cohort (limited by mRNA availability), as described previously (63).

The TCGA BRCA cohort was subsampled (*n* = 269) to match our cohorts. Selection criteria included ER-status positive, HER2-status nonpositive, distant metastasis free, no prior neoadjuvant therapy, and PFS information available (Supplemental Table 12). Samples were divided into a ‘TAM-like’ group of premenopausal patients (*n* = 75) and an ‘AI-like’ group of postmenopausal patients (*n* = 194). Histologic grade was determined using WHO criteria (information on tubule formation, pleomorphism, and cell proliferation from ref. 64).

### Next generation target sequencing and variant calling.

After manual microdissection to enrich for invasive tumor cells, DNA was extracted using the Maxwell RSC DNA FFPE kit on a Maxwell RSC instrument (Promega), and quality control tested (post-QC, discovery cohort, *n* = 362; validation cohort 1, *n* = 222). NGS with 2 gene panels was performed using a S5 prime instrument (ThermoFisher Scientific). The Oncomine Comprehensive v3 assay (ThermoFisher Scientific) covers 161 genes, including copy number variations. The custom-made second panel covers the full-coding sequence of 17 genes frequently mutated in BC (*ABCA13, CBFB, CDH1, ERBB2, ERCC2, ESR1, FAT1, FAT2, FAT3, GATA3, MAP3K1, MUTYH, PIK3CA, RUNX1, RYR2, TBX3,* and *TP53*) (14). Variant calling and functional annotation were performed using ANNOVAR software (65). Deletions and amplifications were selected using a copy number cutoff ≤ 0.6 (upper CI ≤ 0.75) and ≥ 5.0 (lower CI ≥ 3.5), respectively. The results were manually curated to exclude FFPE-related artifacts.

### DNA methylation preprocessing and cell type deconvolution.

DNA (range 84–1,400 ng) was submitted to the DKFZ Genome and Proteome core facility for methylation analysis using HumanMethylationEPIC (EPIC) BeadChips (Illumina), including a restore step for FFPE material. Validation cohort 2 was analyzed using Infinium MethylationEPIC v2.0 BeadChips. We downloaded reference datasets from GEO for 6 immune cell types (GSE110554), human mammary fibroblast, epithelial cells, and endothelial cells (GSE74877) (66), and human ER+ BC cells (MCF7, GSE68379) (67). Raw data were preprocessed using the RnBeads R package version 2.15.1 (68, 69), with beta-mixture quantile normalization and no background correction. We excluded probes directly overlapping a SNP (dbSNP 150, minor allele frequency > 0.01), probes with the last 3 bases in their target sequence overlapping a SNP (minor allele frequency > 0.05), cross-hybridizing probes (70, 71) and non-CpG probes. We estimated cell type proportions with the settings inference.max.cell.type.markers = 100,000, inference.top.cell.type.markers = 500, using the Houseman algorithm (72) implemented in RnBeads. For differential methylation analyses in RnBeads (using age and sample processing as covariates), raw data were preprocessed using the SeSAMe package (73) with linear dye-bias correction and background subtraction using noobsb. For gene annotation, we developed a pipeline (https://github.com/gk-zhang/InfiniumEPICMethylation.hg19/tree/main; commit ID 7d87e7851db75fcbe77ccf9febd4594e8a37ed0f) to map CpG sites to the next transcription start site using hg19 transcript information from “EnsDb.Hsapiens.v75” (74). We performed gene set overrepresentation analyses using hallmark gene sets (75). DMS were annotated with chromatin states using published ChromHMM classification for MCF7 cells (76). A consensus list of PMDs in human BC was constructed from ref. 77, with occurrence in ≥ 9 of 30 tumors and a size >100 kb (*n* = 2,538 regions). We calculated MeTIL scores as described in ref. 23. Illumina HumanMethylation450 data for TCGA BRCA was downloaded from portal.gdc.cancer.gov and preprocessed with RnBeads using scaling.internal for normalization and background subtraction using noobsb. Gene expression HTSeq FPKM-UQ quantification data were downloaded with the function GDCquery of R package TCGAbiolinks (version: 2.22.1) and used to compute Pearson and Spearman correlations between beta values and log_2_ expression values.

### Building of the PERCI.

Predictive indices of endocrine resistance were constructed from discovery cohort features using lasso penalized logistic regression with the R package glmnet (version 4.1–3). Internal cross validation was used to determine model hyperparameters (78) based on optimizing the quality of out-of-sample prediction in terms of discrimination by ROC-AUC (79). Only samples with complete data were used (Supplemental Table 1). Input data included DMS beta values with mean methylation difference ≥ 10%, RGA (set as 0 for WT and 1 for alterations) with significant differences between response groups (*P* < 0.025), patient age and cell type proportions. The prediction outcome was defined as a binary classification of R and NR. We evaluated the model performance by calculating ROC-AUC between the known classification result and the prediction scores (described below) using the R package pROC (version: 1.18.0). The final models were selected based on the best ROC-AUCs in validation cohort 1.

Each model is composed of predictors and coefficients. PERCI TAM and AI scores were calculated as the weighted sum of the predictor values multiplied by the model coefficients. PERCI scores were scaled to a range (from 0 to 1) by dividing the original score by the difference between the theoretical maximum score and the theoretical minimum score, obtained by assigning a (beta) value of 1/0 to CpG sites or mutated genes with positive coefficients and 0/1 to those with negative coefficients. For patient age and cell type information, the median values of the discovery cohort data were used for the calculation of both theoretical maximum and minimum score.

Accuracy, precision (equal to the positive predictive value), and recall from the pROC output were used to evaluate the performance of the prediction models. We defined the scaled PERCI score with the highest accuracy as a treatment-specific cut off for PERCI low and high groups. F1 score was calculated as *2 × precision × recall/(precision + recall)*. In the confusion matrix, responders are defined as controls and nonresponders as cases.

### Constructing PERCI 450k.

PERCI 450k models and scaled scores were constructed as described above, using only patient age and CpG sites covered on the 450k array, with a mean methylation difference ≥ 5% and *P* values in a range of *P* ≤ 0.05 to *P* ≤ 0.00005. In the TCGA BRCA subcohort, the 450k models were evaluated based on their ability to predict PFS. Survival analyses were performed with the R package survival (version 3.5–0), using information on time to progression or censoring within 10 years. Patients were classified into PERCI 450k high and low groups using cutoffs chosen by minimizing the *P* value of the log-rank test.

### Survival analyses of the WGS-ADAPT trial subcohorts.

Survival analyses were conducted to predict invasive disease-free survival (IDFS), distant disease-free survival (DDFS), and overall survival (OS). IDFS is defined as the time interval from randomization until invasive local and/or regional relapse, invasive contralateral BC, invasive second malignancy, distant relapse, or death by any cause; DDFS as the time interval from randomization until distant events comprise distant relapse or death by any cause and OS as the time interval from randomization until death by any cause.

To estimate the relationship parameters between PERCI and survival outcomes, PERCI (original and scaled versions) was used as a continuous variable with univariate Cox Proportional Hazards models or as a dichotomized variable based on treatment-specific cut off values. As PERCI scores are treatment specific, the models considered the treatment-specific subcohorts, and analyses were stratified for the discovery and validation cohorts. Risk differences between groups with lower and higher PERCI score values were analyzed using Kaplan–Meier plots. Log-rank tests were performed for between-group significance tests. Significance levels for all statistical tests was 5% and no adjustments for multiple testing were performed.

### Statistics.

In the discovery cohort with matched design, McNemar’s test for symmetry was used to compare R and NR patients for clinical variables. In the validation cohort, the chi-squared test for trends was used to compare Ki67 at baseline and Ki67 post-pET, and Fisher’s exact test was used for all other comparisons. The relationship between clinical variables and TAM and AI prediction scores was calculated using Spearman’s rank correlation coefficient.

Fisher’s exact test was used to compare RGA frequencies between responders and nonresponders in each treatment group (minimum 7.5% RGA frequency in at least one of the subgroups (TAM, AI, NR, R) in the discovery or validation cohort 1), and to test the significant difference of clinico-pathological variables between the PERCI 450k score high and low groups (Supplemental Table 12). *P* values < 0.05 were considered significant unless stated otherwise. Wilcoxon test was used to compare PERCI/PERCI 450k between TAM/AI responders and nonresponders per RS groups for all 3 cohorts (paired in the discovery cohort).

### Study approval.

The study design is following the guidelines of the local ethics committee. The study was approved by the ethics committee of the Medical School Hannover (Hannover, Germany) with reference ID 2716-2015 on August 22, 2017. Written consent was obtained by the participants.

### Data availability.

DNA methylation data was uploaded to GEO with accession number GSE261142 (discovery cohort, validation cohort 1) and GSE274415 (validation cohort 2). NanoString RNA expression data is accessible under GSE277607. NGS panel sequencing data is available from EGA under accession number EGAD50000001595. Access to survival data from the WSG-ADAPT trial is restricted. Values for all data points in graphs are reported in the Supporting Data Values file.

## Author contributions

HHK, NH, UM, and MC designed the study. OG, MG, SK, UN, and NH collected patient samples and data. REK and MC selected the patients and performed the matching. MC, HC, LDK, and MR performed the immunohistochemical staining and pathology assessment. SB, GZ, CG, VJ, DS, UL, WH, UM, LM, CZE, HN, ALK, and JK performed the experiments, analyzed, and interpreted the data. UM provided supervision. GZ and CG wrote the manuscript. SB, MC, HK, CP, NH, and UM reviewed and edited the manuscript. All authors read and approved the final manuscript.

## Supplementary Material

Supplemental data

ICMJE disclosure forms

Supplemental table 1

Supplemental table 6

Supplemental table 7

Supplemental table 8

Supplemental table 13

Supporting data values

## Figures and Tables

**Table 1:**
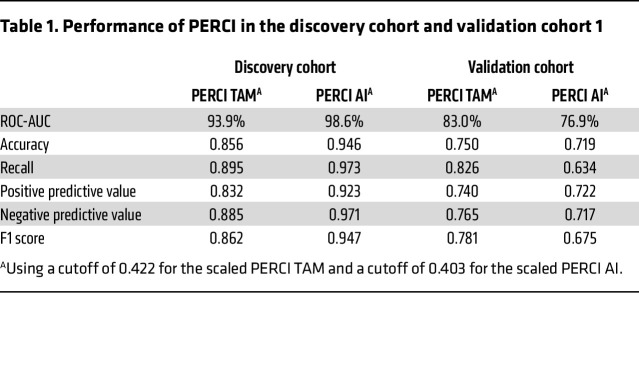
Performance of PERCI in the discovery cohort and validation cohort 1

**Table 2:**
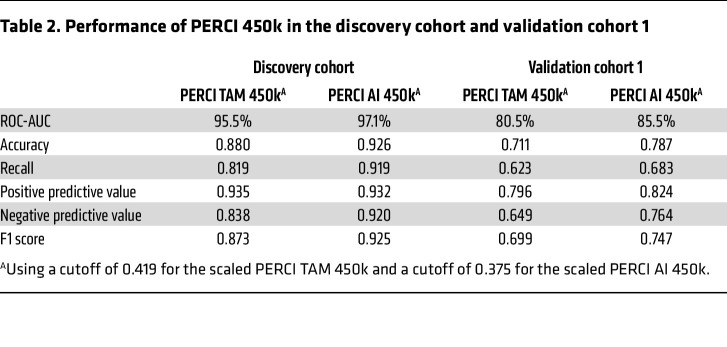
Performance of PERCI 450k in the discovery cohort and validation cohort 1

**Table 3:**
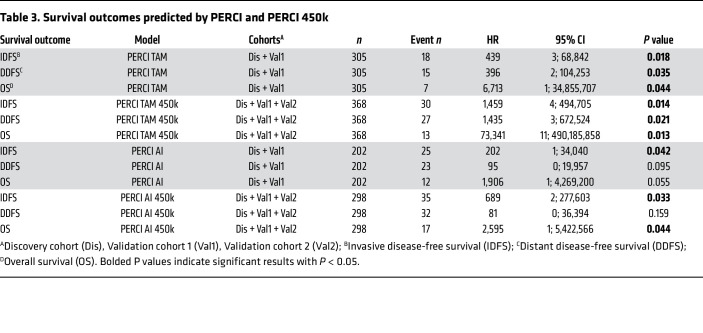
Survival outcomes predicted by PERCI and PERCI 450k

## Version 1 07/24/2025

In-Press Preview

## Version 2 10/01/2025

Electronic publication
