# Supplementary material for: A predictive endocrine resistance index accurately stratifies luminal breast cancer treatment responders and nonresponders
Source: J Clin Invest. 2025 Jul 24;135(19):e177813. doi: 10.1172/JCI177813 (PMC12483570; doi:10.1172/JCI177813)
Supplement: Supplemental data [file jci-135-177813-s078.pdf]

Supplemental information for

A predictive endocrine resistance index accurately stratifies luminal breast cancer treatment responders and non-responders

Guokun Zhang[1], Vindi Jurinovic[1], Stephan Bartels[2], Matthias Christgen[2], Henriette Christgen[2], Leonie Donata Kandt[2], Lidiya Mishieva[3,4], Hua Ni[5], Mieke Raap[2], Janin Klein[6], Anna-Lena Katzke[6,] Winfried Hofmann[6], Doris Steinemann[6], Ronald E. Kates[7], Oleg Gluz[7,8,9], Monika Graeser[7,8,10], Sherko Kümmel[7,11,12], Ulrike Nitz[7,8], Christoph Plass[13], Ulrich Lehmann[2], Christine zu Eulenburg[7,14], Ulrich Mansmann[1], Clarissa Gerhäuser[13*], Nadia Harbeck[5,7], Hans H. Kreipe[2,7]

[1]Institute for Medical Information Processing, Biometry, and Epidemiology, Medical Faculty, Ludwig Maximilians University (LMU), Munich, Germany
[2]Institute of Pathology, Hannover Medical School, Hannover, Germany
[3]Institute of Sociology (IfS), Faculty of Social Sciences, University of Bremen, Bremen, Germany
[4]Department of Methodology and Statistics, Faculty of Social & Behavioural Sciences, Utrecht University, Utrecht, The Netherlands
[5]Breast Center, Department OB&GYN and CCC Munich, LMU University Hospital, Munich, Germany
[6]Department of Human Genetics, Hannover Medical School, Hannover, Germany
[7]West German Study Group (WSG), Moenchengladbach, Germany
[8]Ev. Bethesda Hospital, Breast Center Niederrhein, Moenchengladbach, Germany
[9]University Clinics Cologne, Women's Clinic and Breast Center, Cologne, Germany
[10]Department of Gynecology, University Medical Center Hamburg, Germany
[11]Clinics Essen-Mitte, Breast Unit, Essen, Germany
[12]Charité - Universitätsmedizin Berlin, Department of Gynecology with Breast Center, Berlin, Germany
[13]Division of Cancer Epigenomics, German Cancer Research Center (DKFZ), Heidelberg, Germany
[14]Department of Biometry and Epidemiology, University Medical Center Hamburg, Hamburg, Germany

Present address HN: Department of Pathology, School of Basic Medical Sciences, Peking University Third Hospital, Peking University Health Science Center

*Corresponding author:
Dr. Clarissa Gerhäuser, Division of Cancer Epigenomics, German Cancer Research Center, Im Neuenheimer Feld 280, 69120 Heidelberg, Germany
Phone: +40 6221 423306, email: c.gerhauser@dkfz.de

Supplemental Figure 1. Endocrine therapy-induced changes in histologic grade per treatment group in the discovery and validation cohorts.

Supplemental Figure 2. Correlations of quantitative clinical features with TME components, PERCI scores and recurrent genomic alterations.
Spearman correlation coefficients were calculated between selected features in the discovery and validation cohort 1 for the TAM and AI groups.

Supplemental Figure 3. Correlations of categorical clinical features with TME components, PERCI scores and recurrent genomic alterations.
Kendall correlation coefficients were calculated between selected features in the discovery and validation cohort 1 for the TAM and AI groups.

Supplemental Figure 4. pET resistance-related alterations in the methylome and gene expression.
Density plots of mean methylation beta values of responder vs. non-responder groups for TAM- and AI-treated cases in the discovery and the validation cohort. Percentage of DMS, split in hypo- and hypermethylated in NR, overlapping with chromatin regions derived from ChromHMM analyses of MCF7 cells. Gene set enrichment analyses. Examples of differentially methylated CpG sites correlating with gene expression and PERCI 450k.

Supplemental Figure 5. pET resistance-related alterations in the tumor microenvironment.
Distributions of major immune cell fractions in responder vs. non-responder groups for TAM- and AI-treated cases in the discovery and the validation cohort 1. Spearman correlation of stromal TILs from H&E-stained slides with methylation-derived TILs.

Supplemental Figure 6. ROC AUC of all predictors of PERCI TAM.
Area under the receiver operating characteristic curve (AUC) of the individual predictors of PERCI TAM in the TAM discovery cohort.

Supplemental Figure 7. ROC AUC of all predictors of PERCI AI.
Area under the receiver operating characteristic curve (AUC) of the individual predictors of PERCI AI in the AI discovery cohort.

Supplemental Figure 8. Performance of PERCI TAM 450k and PERCI AI 450k in the discovery cohort and validation cohort 1.
Heatmap of z-scores of age and methylation of CpG sites selected to build PERCI TAM 450k and PERCI AI 450k. Area under receiver operating characteristic curve (AUC) analysis of PERCI 450k's

performance in the discovery cohort and validation cohort 1. Boxplots to stratify PERCI 450k by RS groups and response in the discovery cohort and validation cohort 1.

Supplemental Figure 9. Recurrent genomic alterations (RGA) in the TCGA BRCA sub-cohort.
Oncoprint of recurrent genomic alterations in the TCGA BRCA sub-cohort, color-coded by the mutational event type and separated into high and low PERCI 450k, sorted by total alteration burden.

Supplemental Table 1 (xlsx). Clinico-pathologic data of the discovery and validation cohorts and detailed description of the Predictive Endocrine ResistanCe Index (PERCI) and PERCI 450k.
Clinico-pathological information, cell type composition, data availability indicator in the discovery and the validation cohort. Features, coefficients and data of PERCI TAMl, PERCI TAM 450k, PERCI AI and PERCI AI 450k, PERCI scores.

Supplemental Table 2 (doc). Clinico-pathologic characteristics of the discovery cohort.

Supplemental Table 3 (doc). Clinico-pathologic characteristics of the validation cohort 1.

Supplemental Table 4 (doc). Clinico-pathologic characteristics of the validation cohort 2.

Supplemental Table 5 (doc). Comparison of discovery and validation cohort 1 at baseline

Supplemental Table 6 (xlsx). Detailed information of the NGS panel sequencing.
Sequencing information, and details of mutations and copy number status for cases of the discovery and the validation cohorts.

Supplemental Table 7 (xlsx). Detailed information of the differentially methylated CpG sites (DMS).
IDs, genomic positions, means per TAM and AI groups, statistic, annotation to genes and chromatin regions (MCF7), expression of annotated genes in TCGA-BRCA sub-cohort, pearson and spearman correlation coefficients and p-values, beta values for TAM DMS and AI DMS in the discovery and the validation cohorts and in the TCGA-BRCA sub-cohort (overlap with 450k), RNA expression values (log2 FPKM-UQ) of the annotated genes in the TCGA-BRCA sub-cohort.

Supplemental Table 8 (xlsx). Nanostring BC360 gene expression data for selected genes

Supplemental Table 9 (doc). AUC of individual clinico-pathological characteristics in discovery cohort

Supplemental Table 10 (doc). AUC of individual clinico-pathological characteristics in validation cohort 1

Supplemental Table 11 (doc). AUC of individual clinico-pathological characteristics in validation cohort 2

Supplemental Table 12 (doc). Clinico-pathologic features of the TCGA-BRCA sub-cohort.

Supplemental Table 13 (xlsx). Clinico-pathological data of the TCGA-BRCA sub-cohort and PERCI 450k prediction models. Clinico-pathologic information. Features, coefficients and data of PERCI TAM 450k and PERCI AI 450k model in the TCGA-BRCA sub-cohort and PERCI 450k scores.

Supplemental Table 14 (doc). AUC of individual clinico-pathological characteristics in the TCGA-BRCA subcohort

Supporting data values (xls). data values for all graphs, and values behind any reported means in the manuscript or supplement.

Supplemental Tables 2, 3, 4, 5, 9, 10, 11, 12, 14 are included in this .doc file.

Supplemental Tables

Supplemental Table 2. Clinico-pathologic characteristics of the discovery cohort

Features	ALL	TAM Responder[A]	TAM Non-Responder[B]	AI Responder[A]	AI Non-Responder[B]	P_{TAM}	P_{AI}
Numbers	**n=364**	**n=107**	**n=107**	**n=75**	**n=75**		
	n (%)	n (%)	n (%)	n (%)	n (%)		
age						***0.029***	***9.0e-06***
< 50	116 (32%)	49 (46%)	62 (58)	0	5 (7%)		
50 - 59	133 (36%)	47 (44%)	43 (40%)	9 (12%)	34 (45%)		
≥ 60	115 (32%)	11 (10%)	2 (2%)	66 (88%)	36 (48%)		
histology, baseline						*NA*	*NA*
NST	324 (89%)	92 (86%)	92 (86%)	70 (93%)	70 (93%)		
ILBC	40 (11%)	15 (14%)	15 (14%)	5 (7%)	5 (7%)		
pT stage						*NA*	*NA*
pT1	244 (67%)	71 (66%)	71 (66%)	51 (68%)	51 (68%)		
pT2	118 (32%)	35 (33%)	35 (33%)	24 (32%)	24 (32%)		
pT3	2 (1%)	1 (1%)	1 (1%)	0	0		
pT4	0	0	0	0	0		
pN stage						*NA*	*NA*
pN0	342 (94%)	102 (95%)	102 (95%)	69 (92%)	69 (92%)		
pN1+	22 (6%)	5 (5%)	5 (5%)	6 (8%)	6 (8%)		
grade, baseline						*NA*	*NA*
G1	20 (6%)	7 (7%)	7 (7%)	2 (3%)	2 (3%)		
G2	198 (54%)	73 (68%)	73 (68%)	27 (36%)	27 (36%)		
G3	146 (40%)	27 (25%)	27 (25%)	46 (61%)	46 (61%)		
grade, post-pET						***5.9e-16***	***5.9e-13***
G1	36 (10%)	23 (21%)	0	13 (17%)	0		
G2	214 (59%)	84 (79%)	46 (46%)	62 (83%)	22 (29%)		
G3	114 (31%)	0	61 (57%)	0	53 (71%)		
ER, baseline						*1*	*0.248*
> 10%	358 (98%)	106 (99%)	107 (100%)	74 (99%)	71 (95%)		
≤ 10%	4 (1%)	1 (1%)	0	0	3 (4%)		
n.a.	2 (1%)	0	0	1 (1%)	1 (1%)		
ER, post-pET						*1*	*0.074*
> 10%	358 (98%)	106 (99%)	107 (100%)	75 (100%)	70 (93%)		
≤ 10%	6 (2%)	1 (1%)	0	0	5 (7%)		
PR, baseline						*0.332*	*0.345*
> 10%	305 (84%)	101 (94%)	96 (90%)	57 (76%)	51 (68%)		
≤ 10%	59 (16%)	6 (6%)	11 (10%)	18 (24%)	24 (32%)		
PR, post-pET						*1*	*0.153*
> 10%	246 (68%)	95 (89%)	95 (89%)	24 (32%)	32 (43%)		
≤ 10%	118 (32%)	12 (11%)	12 (11%)	51 (68%)	43 (57%)		
HER2, baseline						*1*[C]	*0.480*[C]

IHC 0/1+	311 (85%)	92 (86%)	90 (84%)	66 (88%)	63 (84%)		
IHC 2+, FISH-neg	49 (13%)	14 (13%)	15 (14%)	8 (11%)	12 (16%)		
IHC 2+, FISH-n.a.	1 (0%)	0	1 (1%)	0	0		
IHC 2+, FISH-pos[D]	3 (1%)	1 (1%)	1 (1%)	1 (1%)	0		
IHC 3+[D]	0	0	0	0	0		
HER2, post-pET						*0.542*[C]	*0.100*[C]
IHC 0/1+	268 (74%)	83 (78%)	77 (72%)	49 (65%)	59 (79%)		
IHC 2+, FISH-neg	95 (26%)	24 (22%)	29 (27%)	26 (35%)	16 (21%)		
IHC 2+, FISH-n.a.	0	0	0	0	0		
IHC 2+, FISH-pos[D]	0	0	0	0	0		
IHC 3+[D]	1 (0%)	0	1 (1%)	0	0		
E-cadherin, baseline						*0.480*[C]	*1*[C]
pos.	317 (87%)	90 (84%)	90 (84%)	68 (91%)	69 (92%)		
neg.	44 (12%)	17 (16%)	15 (14%)	6 (8%)	6 (8%)		
n.a.	3 (1%)	0	2 (2%)	1 (1%)	0		
E-cadherin, post-pET						*0.480*[C]	*0.480*[C]
pos.	314 (86%)	90 (84%)	89 (83%)	67 (89%)	68 (91%)		
neg.	44 (12%)	17 (16%)	15 (14%)	5 (7%)	7 (9%)		
n.a.	6 (2%)	0	3 (3%)	3 (4%)	0		
Ki67, baseline						*NA*	*NA*
0% - 9%	21 (6%)	10 (9%)	7 (7%)	2 (3%)	2 (3%)		
10% - 19%	101 (28%)	31 (29%)	42 (39%)	15 (20%)	13 (17%)		
20% - 34%	205 (56%)	61 (57%)	51 (47%)	44 (58%)	49 (65%)		
35% - 100%	37 (10%)	5 (5%)	7 (7%)	14 (19%)	11 (15%)		
Ki67, post-pET						***3.6e-05***	***2.2e-05***
0% - 9%	182 (50%)	107 (100%)	0	75 (100%)	0		
10% - 19%	0	0	0	0	0		
20% - 34%	143 (39%)	0	88 (82%)	0	55 (73%)		
35% - 100%	39 (11%)	0	19 (18%)	0	20 (27%)		
Luminal subtype[E], baseline						*0.124*	*1*
LumA	268 (74%)	96 (90%)	87 (81%)	43 (57%)	42 (56%)		
LumB	96 (26%)	11 (10%)	20 (19%)	32 (43%)	33 (44%)		
PaTILs[F], baseline						*0.310*[C]	*0.307*[C]
0% - 9%	271 (74%)	78 (73%)	85 (79%)	56 (75%)	52 (69%)		
10% - 40%	77 (21%)	25 (23%)	19 (18%)	15 (20%)	18 (24%)		
41% - 100%	3 (1%)	1 (1%)	0	0	2 (3%)		
n.a.	13 (4%)	3 (3%)	3 (3%)	4 (5%)	3 (4%)		
PaTILs[F], post-pET						*0.710*[C]	*0.472*[C]
0% - 9%	288 (79%)	91 (85%)	88 (82%)	57 (76%)	52 (69%)		
10% - 40%	75 (20%)	16 (15%)	19 (18%)	18 (24%)	22 (29%)		
41% - 100%	1 (1%)	0	0	0	1 (2%)		

PaTILs, baseline vs. post-pET, R						*0.078*[C]	*0.838*[C]
PaTILs, baseline vs. post-pET, NR						*1*[C]	*0.502*[C]
Oncotype DX RS Group, baseline						*NA*	*NA*
1 (0 -11)	78 (21%)	30 (28%)	30 (28%)	9 (12%)	9 (12%)		
2 (12 – 25)	208 (57%)	68 (64%)	68 (64%)	36 (48%)	36 (48%)		
3 (26 – 100)	78 (21%)	9 (8%)	9 (8%)	30 (40%)	30 (40%)		

Unless otherwise stated, the values are given in the format n (%), with n corresponding to the number of patients. The McNemar's chi-squared test for symmetry were used for statistical analysis between the matched pairs of Responder and Non-Responder. Significant differences are highlighted in bold. "NA" for the exact matched pairs therefore the p-value could not be computed.
n.a. not available, ER estrogen receptor, PR progesterone receptor, pET preoperative endocrine therapy, RS recurrence score, LumA Luminal A, lumB Luminal B, TAM tamoxifen, AI aromatase inhibitors.
Case selection of the discovery cohort was done in a way that responders and non-responders were balanced for pT, pN, grade (baseline), histological type (baseline and post-pET), and RS group ("matching" to exclude confounding effects by dissimilar histological grades in R and NR).
A: Responder was defined as post-pET Ki67 < 10% and relative Ki67 decrease of ≤ -70% from baseline to post-pET.
B: Non-responder was defined as post-pET Ki67 of ≥ 20% and relative Ki67 decrease of > -20% from baseline to post-pET.
C: Comparison only between the first two category groups.
D: HER2-positivity in a minor subclone of < 10% of tumor cells; not HER2-positive according to ASCO/CAP-Guidelines (1)
E: Luminal B: Ki67 baseline ≥ 35% or PR baseline ≤ 20%, Luminal A: all others.
F: PaTILs: Stromal tumor-infiltrating lymphocytes in pathologic tissue sections.

Supplemental Table 3. Clinico-pathologic characteristics of validation cohort 1

Features	ALL	TAM Responder[A]	TAM Non-Responder[B]	AI Responder[A]	AI Non-Responder[B]	P_{TAM}	P_{AI}
Numbers	**n=270**	**n=75**	**n=80**	**n=67**	**n=48**		
	n (%)	n (%)	n (%)	n (%)	n (%)		
age						***2.4e-03***	*0.692*
< 50	97 (36%)	35 (47%)	59 (74%)	2 (3%)	1 (2%)		
50 – 59	83 (31%)	34 (45%)	18 (22%)	16 (24%)	15 (31%)		
≥ 60	90 (33%)	6 (8%)	3 (4%)	49 (73%)	32 (67%)		
histology, baseline						***3.0e-07***	*0.666*
NST	222 (82%)	48 (64%)	77 (96%)	57 (85%)	40 (85%)		
ILBC	41 (15%)	22 (29%)	2 (3%)	10 (15%)	7 (15%)		
Others[C]	7 (3%)	5 (7%)	1 (1%)	0	1 (2%)		
pT stage						***0.017***	*0.816*
pT1	145 (54%)	43 (57%)	39 (49%)	38 (57%)	25 (52%)		
pT2	113 (42%)	27 (36%)	40 (50%)	26 (39%)	20 (42%)		
pT3	8 (3%)	5 (7%)	0	2 (3%)	1 (2%)		
pT4	4 (1%)	0	1 (1%)	1 (1%)	2 (4%)		
pN stage						*0.390*	*0.206*
pN0	226 (84%)	60 (80%)	69 (86%)	59 (88%)	38 (79%)		
pN1+	44 (16%)	15 (20%)	11 (14%)	8 (12%)	10 (21%)		
grade, baseline						***7.5e-04***	***1.6e-03***
G1	22 (8%)	14 (19%)	4 (5%)	3 (4%)	0		
G2	143 (53%)	51 (68%)	48 (60%)	33 (49%)	11 (23%)		
G3	106 (39%)	10 (13%)	28 (35%)	31 (47%)	37 (77%)		
grade, post-pET						***5.2e-23***	***3.2e-17***
G1	33 (12%)	16 (21%)	0	17 (25%)	0		
G2	146 (54%)	58 (77%)	24 (30%)	49 (73%)	15 (31%)		
G3	91 (34%)	1 (1%)	56 (70%)	1 (2%)	33 (69%)		
ER, baseline						*0.497*	*1*
> 10	266 (99%)	75 (100%)	78 (98%)	66 (99%)	47 (98%)		
≤ 10	4 (1%)	0	2 (2%)	1 (1%)	1 (2%)		
ER, post-pET						*0.611*	*1*
> 10	266 (99%)	73 (97%)	79 (99%)	66 (99%)	48 (100%)		
≤ 10	4 (1%)	2 (3%)	1 (1%)	1 (1%)	0		
PR, baseline						*0.133*	***0.012***
> 10	227 (84%)	72 (96%)	71 (89%)	55 (82%)	29 (60%)		
≤ 10	43 (12%)	3 (4%)	9 (11%)	12 (18%)	19 (40%)		
PR, post-pET							
> 10	167 (62%)	67 (89%)	69 (86%)	23 (34%)	8 (17%)	*0.629*	*0.054*
≤ 10	103 (38%)	8 (11%)	11 (14%)	44 (66%)	40 (83%)		
HER2, baseline							
IHC 0/1+	242 (90%)	68 (91%)	72 (90%)	59 (88%)	43 (90%)	*1*[D]	*1*[D]
IHC 2+, FISH-neg	28 (10%)	7 (9%)	8 (10%)	8 (12%)	5 (10%)		

IHC 2+, FISH-n.a.	0	0	0	0	0		
IHC 2+, FISH-pos[E]	0	0	0	0	0		
IHC 3+[E]	0	0	0	0	0		
HER2, post-pET						*0.573* [D]	*0.371* [D]
IHC 0/1+	208 (77%)	56 (75%)	63 (79%)	54 (81%)	35 (73%)		
IHC 2+, FISH-neg	62 (23%)	19 (25%)	17 (21%)	13 (19%)	13 (27%)		
IHC 2+, FISH-n.a.	0	0	0	0	0		
IHC 2+, FISH-pos[E]	0	0	0	0	0		
IHC 3+[E]	0	0	0	0	0		
E-cadherin, baseline						***1.2e-05***[D]	*1* [D]
pos.	218 (81%)	50 (67%)	75 (94%)	55 (82%)	38 (79%)		
neg.	44 (16%)	22 (29%)	3 (4%)	11 (16%)	8 (17%)		
n.a.	8 (3%)	3 (4%)	2 (2%)	1 (2%)	2 (4%)		
E-cadherin, post-pET						***1.7e-04*** [D]	*0.446* [D]
pos.	218 (81%)	50 (67%)	73 (91%)	53 (79%)	42 (88%)		
neg.	49 (18%)	24 (32%)	6 (8%)	13 (19%)	6 (12%)		
n.a.	3 (1%)	1 (1%)	1 (1%)	1 (2%)	0		
Ki67, baseline						***7.5e-09***	***1.3e-06***
0-9	22 (8%)	12 (16%)	0	10 (15%)	0		
10-19	103 (38%)	52 (69%)	36 (45%)	13 (19%)	2 (4%)		
20-34	83 (31%)	10 (13%)	35 (44%)	25 (37%)	12 (25%)		
35-100	63 (23%)	1 (1%)	9 (11%)	19 (28%)	34 (71%)		
Ki67, post-pET						***6.7e-33***	***1.0e-25***
0-9	142 (53%)	75 (100%)	0	67 (100%)	0		
10-19	0	0	0	0	0		
20-34	87 (32%)	0	46 (58%)	0	41 (85%)		
35-100	41 (15%)	0	34 (42%)	0	7 (15%)		
Luminal subtype[F], baseline						***6.4e-03***	***2.1e-06***
LumA	175 (65%)	70 (93%)	62 (78%)	37 (55%)	6 (13%)		
LumB	95 (35%)	5 (7%)	18 (22%)	30 (44%)	42 (87%)		
PaTILs[G], baseline						***0.019*** [D]	*0.067* [D]
0% - 9%	201 (74%)	62 (83%)	55 (69%)	52 (78%)	32 (67%)		
10% - 40%	59 (22%)	10 (13%)	24 (30%)	10 (15%)	15 (31%)		
41% - 100%	4 (2%)	1 (1%)	1 (1%)	1 (1%)	1 (2%)		
n.a.	6 (2%)	2 (3%)	0	4 (6%)	0		
PaTILs[G] post-pET						***3.7e-03*** [D]	***2.6e-03*** [D]
0% - 9%	223 (82%)	70 (93%)	61 (76%)	61 (91%)	31 (65%)		
10% - 40%	45 (16%)	5 (7%)	19 (24%)	6 (9%)	15 (31%)		
41% - 100%	1 (1%)	0	0	0	1 (2%)		
n.a.	1 (1%)	0	0	0	1 (2%)		

PaTILs, baseline vs. post-pET, R						*0.114* [D]	*0.343* [D]
PaTILs, baseline vs. post-pET, NR						*0.345* [D]	*1* [D]
Oncotype DX RS Group, baseline						***1.9e-08***	***2.1e-12***
1 (0 -11)	57 (21%)	31 (41%)	5 (6%)	20 (30%)	1 (2%)		
2 (12 – 25)	156 (58%)	41 (55%)	56 (70%)	43 (64%)	16 (33%)		
3 (26 – 100)	57 (21%)	3 (4%)	19 (24%)	4 (6%)	31 (65%)		

Unless otherwise stated, the values are given in the format n (%), with n corresponding to the number of patients. For the statistical analysis between the Responder and Non-Responder groups (non-matched), the chi-squared test for trends was used to compare Ki67 at baseline and Ki67 post-pET, and Fisher's exact test was used for all other comparisons. Significant differences are highlighted in bold. “NA” indicates data with clear separation therefore the p value is not available.

n.a. not available, ER estrogen receptor, PR progesterone receptor, pET preoperative endocrine therapy, RS recurrence score, LumA Luminal A, lumB Luminal B, TAM tamoxifen, AI aromatase inhibitors.

Case selection criteria in the validation cohort were made less strict and no matching between NR and R.

A: Responder was defined as post-pET Ki67 <10%.

B: Non-responder was defined as post-pET Ki67 of ≥20%.

C: Others: Tubulary, Medullary, Mucinous.

D: Comparison only between the first two category groups.

E: HER2-positivity in a subclone <10% of tumor cells.

F: Luminal B: Ki67 baseline ≥ 35% or PR baseline ≤ 20%, Luminal A: all others.

G: PaTILs: Stromal tumor-infiltrating lymphocytes in pathologic tissue sections.

Supplemental Table 4. Clinico-pathologic characteristics of validation cohort 2

	ALL	TAM		AI			
		Responder[A]	Non-Responder[B]	Responder[A]	Non-Responder[B]		
	n=176	n=51	n=18	n=92	n=15	P_{TAM}	P_{AI}
	n (%)	n (%)	n (%)	n (%)	n (%)		
age						***3.4e-09***	***2.3e-11***
< 50	39 (22%)	4 (8%)	14 (78%)	7 (8%)	14 (93%)		
50 – 59	43 (25%)	17 (33%)	4 (22%)	22 (24%)	0		
≥ 60	94 (53%)	30 (59%)	0	63 (68%)	1 (7%)		
cT stage						*0.118*	*0.920*
cT1	88 (50%)	25 (49%)	5 (28%)	49 (53%)	9 (60%)		
cT2	76 (43%)	22 (43%)	13 (72%)	35 (38%)	6 (40%)		
cT3	10 (6%)	4 (8%)	0	6 (7%)	0		
cT4	1 (1%)	0	0	1 (1%)	0		
n.a.	1 (1%)	0	0	1 (1%)	0		
cN stage						*0.436*	*0.702*
cN0	154 (87%)	45 (88%)	14 (78%)	82 (89%)	13 (87%)		
cN1+	21 (12%)	6 (12%)	4 (22%)	9 (10%)	2 (13%)		
n.a.	1 (1%)	0	0	1 (1%)	0		
ER, baseline							
> 10	175 (99%)	51 (100%)	18 (100%)	91 (99%)	15 (100%)	*1*	*1*
≤ 10	1 (1%)	0	0	1 (1%)	0		
ER, post-pET							
> 10	176(100%)	51 (100%)	18 (100%)	92 (100%)	15 (100%)	*1*	*1*
≤ 10	0	0	0	0	0		
PR, baseline						*0.667*	*0.651*
> 10	158 (90%)	45 (88%)	17 (94%)	83 (90%)	13 (87%)		
≤ 10	18 (10%)	6 (12%)	1 (6%)	9 (10%)	2 (13%)		
PR, post-pET						*0.101*	*0.104*
> 10	99 (56%)	28 (55%)	14 (78%)	46 (50%)	11 (73%)		
≤ 10	77 (44%)	23 (45%)	4 (22%)	46 (50%)	4 (27%)		
HER2, baseline						*1*[C]	*0.457*[C]
IHC 0/1+	150 (85%)	44 (86%)	16 (89%)	76 (83%)	14 (93%)		
IHC 2+, FISH-neg	26 (15%)	7 (14%)	2 (11%)	16 (17%)	1 (7%)		
IHC 2+, FISH-n.a.	0	0	0	0	0		
IHC 2+, FISH-pos[D]	0	0	0	0	0		
IHC 3+[D]	0	0	0	0	0		
HER2, post-pET						*1*[C]	*0.773*[C]
IHC 0/1+	120 (68%)	34 (67%)	12 (67%)	64 (70%)	10 (67%)		
IHC 2+, FISH-neg	56 (32%)	17 (33%)	6 (33%)	28 (30%)	5 (33%)		
IHC 2+, FISH-n.a.	0	0	0	0	0		

IHC 2+, FISH-pos[D]	0	0	0	0	0		
IHC 3+[D]	0	0	0	0	0		
E-cadherin, baseline						*0.401*[C]	*0.183*[C]
pos.	121 (69%)	29 (57%)	13 (72%)	65 (71%)	14 (93%)		
neg.	49 (28%)	20 (39%)	5 (28%)	23 (25%)	1 (7%)		
n.a.	9 (3%)	2 (4%)	0	4 (4%)	0		
E-cadherin, post-pET						*0.399*[C]	*0.179*[C]
pos.	119 (68%)	29 (57%)	13 (72%)	63 (68%)	14 (93%)		
neg.	48 (27%)	21 (41%)	5 (28%)	21 (23%)	1 (7%)		
n.a.	9 (5%)	1 (2%)	0	8 (9%)	0		
Ki67, baseline						***1.7e-08***	***2.8e-06***
0-9	21 (12%)	7 (14%)	0	14 (15%)	0		
10-19	72 (41%)	25 (49%)	1 (5%)	45 (49%)	1 (7%)		
20-34	61 (35%)	19 (37%)	5 (28%)	29 (32%)	8 (53%)		
35-100	22 (12%)	0	12 (67%)	4 (4%)	6 (40%)		
Ki67, post-pET						***3.8e-16***	***2.1e-24***
0-9	143 (81%)	51 (100%)	0	92 (100%)	0		
10-19	0	0	0	0	0		
20-34	28 (16%)	0	15 (83%)	0	13 (87%)		
35-100	5 (3%)	0	3 (17%)	0	2 (13%)		
Luminal subtype[E], baseline						***2.3e-04***	*0.078*
LumA	133 (76%)	42 (82%)	6 (33%)	76 (83%)	9 (60%)		
LumB	43 (24%)	9 (18%)	12 (67%)	16 (17%)	6 (40%)		
Oncotype DX RS Group, baseline						***4.5e-06***	***5.5e-06***
1 (0 -11)	53 (30%)	23 (45%)	0	30 (33%)	0		
2 (12 – 25)	101 (57%)	26 (51%)	10 (56%)	57 (62%)	8 (53%)		
3 (26 – 100)	20 (11%)	2 (4%)	8 (44%)	3 (3%)	7 (47%)		
n.a.	2 (1%)	0	0	2 (2%)	0		

Unless otherwise stated, the values are given in the format n (%), with n corresponding to the number of patients. For the statistical analysis between the Responder and Non-Responder groups (non-matched), the chi-squared test for trends was used to compare Ki67 at baseline and Ki67 post-pET, and Fisher's exact test was used for all other comparisons. Significant differences are highlighted in bold. "NA" indicates data with clear separation therefore the p value is not available.

n.a. not available, ER estrogen receptor, PR progesterone receptor, pET preoperative endocrine therapy, RS recurrence score, LumA Luminal A, lumB Luminal B, TAM tamoxifen, AI aromatase inhibitors.

Case selection criteria in the validation cohort were made less strict and no matching between NR and R.

A: Responder was defined as post-pET Ki67 <10%.

B: Non-responder was defined as post-pET Ki67 of ≥20%.

C: Comparison only between the first two category groups.

D: HER2-positivity in a subclone <10% of tumor cells.

E: Luminal B: Ki67 baseline ≥ 35% or PR baseline ≤ 20%, Luminal A: all others.

Supplemental Table 5. Comparison of discovery and validation cohort 1 at baseline

Parameters[A]	$P_{TAM\ Responder}$	$P_{TAM\ Non\text{-}Responder}$	$P_{AI\ Responder}$	$P_{AI\ Non\text{-}Responder}$
age	*0.926*	***0.027***	***0.036***	*0.119*
histology type	***3.3e-04***	***0.006***	*0.170*	*0.119*
pT	*0.085*	***0.021***	*0.171*	***0.045***
pN	***0.002***	***0.035***	*0.575*	*0.054*
grade	***0.014***	*0.356*	*0.194*	*0.139*
ER	*1.000*	*0.182*	*0.475*	*1.000*
PR	*0.738*	*1.000*	*0.415*	*0.441*
HER2	*0.486*	*0.501*	*1.000*	*0.434*
E-cadherin	***0.026***	***0.023***	*0.194*	*0.147*
Ki67	***9.3e-10***	*0.062*	***0.011***	***1.8e-09***
luminal subtype	*0.439*	*0.583*	*0.866*	***1.3e-06***
Oncotype DX RS Group	*0.119*	***4.0e-05***	***1.9e-06***	*0.011*

For the statistical analysis between the cohorts (separately by treatment and response groups), Fisher's exact test was used. Significant differences with $P < 0.05$ are highlighted in bold.

ER estrogen receptor, PR progesterone receptor, RS recurrence score, TAM tamoxifen, AI aromatase inhibitors.

A: for details of group descriptions, see Supplemental Tables 2 and 3.

Supplemental Table 9. AUC of individual clinico-pathological characteristics in the discovery cohort

Parameters	**AUC** TAM	**AUC** AI
Age	61.9% (54.4%-69.3%)	69.8% (61.2%-78.5%)
Histology type baseline[A]	50.0% (45.3%-54.7%)	50.0% (46.0%-54.0%)
pT[A]	50.0% (43.6%-56.4%)	50.0% (42.5%-57.5%)
pN[A]	50.0% (47.2%-52.8%)	50.0% (45.6%-54.4%)
Histology grade baseline[A]	50.0% (43.7%-56.3%)	50.0% (42.1%-57.9%)
ER percent baseline	58.5% (51.4%-65.6%)	42.4% (34.4%-50.4%)
PR percent baseline	45.8% (38.3%-53.3%)	46.4% (37.2%-55.7%)
HER2 IHC Score baseline	46.2% (39.1%-53.3%)	47.4% (39.1%-55.7%)
E-Cadherin Status baseline[A]	50.8% (46.0%-55.6%)	50.1% (45.7%-54.5%)
Ki67 baseline	50.0% (42.4%-57.7%)	49.7% (40.6%-58.8%)
Oncotype DX RS Group	50.0% (43.4%-56.6%)	50.0% (41.6%-58.4%)
Luminal subtype[A]	54.2% (49.5%-58.9%)	50.7% (42.7%-58.6%)

ROC-AUC (with 95% confidence intervals) of individual clinic-pathological characteristics to predict pET response in the discovery cohort.

ER estrogen receptor, PR progesterone receptor, pET preoperative endocrine therapy, RS recurrence score, TAM tamoxifen, AI aromatase inhibitors.

A: Categorical variables were converted to numerical values for the calculation of AUC.

Supplemental Table 10. AUC of individual clinico-pathological characteristics in validation cohort 1

**Parameters**	**AUC** TAM	**AUC** AI
Age	66.2% (57.6%-74.9%)	57.7% (47.2%-68.2%)
Histology type baseline[A]	60.6% (54.1%-67.1%)	51.1% (44.2%-57.9%)
pT[A]	52.9% (44.8%-61.0%)	52.6% (43.1%-62.2%)
pN[A]	47.0% (41.1%-53.0%)	54.3% (47.3%-61.3%)
Histology grade baseline[A]	64.7% (57.6%-71.9%)	65.9% (57.5%-74.3%)
ER percent baseline	44.8% (36.1%-53.5%)	44.0% (34.5%-53.5%)
PR percent baseline	59.2% (50.4%-68.0%)	70.5% (60.8%-80.1%)
HER2 IHC Score baseline	47.3% (39.1%-55.5%)	54.9% (45.3%-64.5%)
E-Cadherin Status baseline[A]	64.0% (58.2%-69.9%)	50.3% (43.0%-57.5%)
Ki67 baseline	69.1% (61.0%-77.2%)	78.5% (70.4%-86.6%)
Oncotype DX RS Group	72.6% (66.2%-79.1%)	83.6% (77.0%-90.2%)
Luminal subtype[A]	57.9% (52.5%-63.3%)	71.4% (63.7%-79.0%)

ROC-AUC (with 95% confidence intervals) of individual clinic-pathological characteristics to predict pET response in the validation cohort.

ER estrogen receptor, PR progesterone receptor, pET preoperative endocrine therapy, RS recurrence score, TAM tamoxifen, AI aromatase inhibitors.

A: Categorical variables were converted to numerical values for the calculation of AUC.

Supplemental Table 11. AUC of individual clinico-pathological characteristics in validation cohort 2

**Parameters**	**AUC** TAM	**AUC** AI
Age	93.8% (88.5%-99.2%)	94.9% (89.9%-99.9%)
cT[A]	57.8% (45.1%-70.5%)	45.4% (32.3%-58.5%)
cN[A]	55.0% (44.3%-65.8%)	52.0% (42.3%-61.7%)
ER percent baseline	62.6% (47.1%-78.1%)	67.0% (51.9%-82.1%)
PR percent baseline	51.3% (35.8%-66.7%)	56.6% (39.9%-73.2%)
HER2 IHC Score baseline	41.7% (27.1%-56.3%)	48.1% (34.9%-61.3%)
E-Cadherin Status baseline[A]	54.3% (41.7%-66.9%)	55.8% (46.5%-65.1%)
Ki67 baseline	94.9% (88.8%-100.0%)	87.9% (79.6%-96.2%)
Oncotype DX RS Group	82.8% (74.6%-91.0%)	80.6% (71.2%-89.9%)
Recurrence Score	86.2% (76.8%-95.7%)	76.4% (63.1%-89.7%)
Luminal subtype[A]	74.5% (62.1%-86.9%)	61.3% (47.9%-74.7%)

ROC-AUC (with 95% confidence intervals) of individual clinic-pathological characteristics to predict pET response in the validation cohort 2.

ER estrogen receptor, PR progesterone receptor, pET preoperative endocrine therapy, RS recurrence score, TAM tamoxifen, AI aromatase inhibitors.

A: Categorical variables were converted to numerical values for the calculation of AUC.

Supplemental Table 12. Clinico-pathologic features of the TCGA-BRCA sub-cohort

	**All**	**TAM-like**		**AI-like**			
		**PERCI TAM 450k low**	**PERCI TAM 450k high**	**PERCI AI 450k low**	**PERCI AI 450k high**		
	**n=269**	**n=25**	**n=50**	**n=143**	**n=51**	$P_{TAM\ like}$	$P_{AI\ like}$
	n (%)	n (%)	n (%)	n (%)	n (%)		
Age						*0.208*	***1.9e-03***
< 50	73 (26%)	18 (72%)	43 (86%)	6 (4%)	6 (12%)		
50 - 59	78 (30%)	7 (28%)	7 (14%)	40 (28%)	24 (47%)		
≤ 60	118 (44%)	0	0	97 (68%)	21 (41%)		
Menopause						*1*	*1*
Pre	75 (28%)	25 (100%)	50 (100%)	0	0		
Post	194 (72%)	0	0	143 (100%)	51 (100%)		
Histology						***0.026***	*0.287*
NST	190 (71%)	14 (56%)	41 (82%)	96 (67%)	39 (76%)		
ILBC	79 (29%)	11 (44%)	9 (18%)	47 (33%)	12 (24%)		
pT Stage						*0.516*	***0.026***
pT1	77 (29%)	3 (12%)	10 (20%)	55 (38%)	9 (18%)		
pT2	156 (58%)	16 (64%)	33 (66%)	72 (50%)	35 (68%)		
pT3	35 (13%)	6 (24%)	7 (14%)	15 (11%)	7 (14)		
pT4	1 (0%)	0	0	1 (1%)	0		
pN Stage						*0.616*	*1*
pN0	126 (47%)	11 (44%)	18 (36%)	71 (50%)	26 (51%)		
pN1+	143 (53%)	14 (56%)	32 (64%)	72 (50%)	25 (49%)		
Grade						*0.367*	***0.026***
G1	74 (27%)	8 (32%)	10 (20%)	49 (34%)	7 (14%)		
G2	123 (46%)	14 (56%)	25 (50%)	59 (41%)	25 (49%)		
G3	57 (21%)	3 (12%)	13 (26%)	27 (19%)	14 (28%)		
NA	15 (6%)	0	2 (4%)	8 (6%)	5 (10%)		
ER						*1*	*1*
Positive	269 (100%)	25 (100%)	50 (100%)	143 (100%)	51 (100%)		
Negative	0	0	0	0	0		
PR						*1*	*0.528*
Positive	228 (85%)	21 (84%)	43 (86%)	123 (86%)	41 (80%)		
Negative	40 (15%)	4 (16%)	7 (14%)	19 (13%)	10 (20%)		
Indeterminate	1 (0%)	0	0	1 (1%)	0		
HER2						*0.289*[A]	*0.107*[A]
Negative	193 (72%)	16 (64%)	38 (76%)	107 (75%)	32 (63%)		
Equivocal[B]	76 (28%)	9 (36%)	12 (24%)	36 (25%)	19 (37%)		
Positive	0	0	0	0	0		
PFS status[C]						***0.026***	*0.084*
0	247 (92%)	25 (100%)	40 (80%)	137 (96%)	45 (88%)		

1	22 (8%)	0	10 (20%)	6 (4%)	6 (12%)

Unless otherwise stated, the values are given in the format n (%), with n corresponding to the number of patients. For the statistical analysis between the TAM/AI score low and TAM/AI score high, the Fisher's exact test (FET) was used. Significant differences are highlighted in bold.

ER estrogen receptor, PR progesterone receptor, PFS progression-free survival.

A: Comparison only between the first two category groups.

B: Her2 equivocal was defined in TCGA either with scores of 2+ in the immunohistochemistry (IHC) assay or with a HER2/CEP17 ratio between 1.8 and 2.2 in Fluorescence in situ hybridization (FISH).

C: 0 censored, 1 progression

Supplemental Table 14. AUC of individual clinico-pathological characteristics in the TCGA-BRCA sub-cohort

**Parameters**	**AUC** TAM-like	**AUC** AI-like
Age	73.6% (62.4%-84.7%)	65.2% (56.5%-73.9%)
Histology type[A]	63.0% (51.7%-74.3%)	54.7% (47.6%-61.7%)
pT[A]	42.6% (30.8%-54.3%)	60.0% (52.5%-67.5%)
pN[A]	54.0% (42.0%-66.0%)	50.7% (42.6%-58.7%)
Grade[A]	60.0% (47.8%-72.3%)	61.8% (53.5%-70.0%)
PR status[A]	51.0% (42.2%-59.8%)	47.3% (41.1%-53.4%)
HER2 status[A]	56.0% (44.7%-67.3%)	44.0% (36.4%-51.6%)
PFS status[A]	60.0% (54.4%-65.6%)	53.8% (49.0%-58.5%)

ROC-AUC (with 95% confidence intervals) of individual clinic-pathological characteristics to predict TAM/AI low and high scores in the TCGA-BRCA sub-cohort. Cases were classified as TAM-like and AI-like by menopausal status.
ER estrogen receptor, PR progesterone receptor, PFS progression-free survival
A: Categorical variables were converted to numerical values for the calculation of AUC.

Additional references

1. Wolff AC, et al. Recommendations for human epidermal growth factor receptor 2 testing in breast cancer: American Society of Clinical Oncology/College of American Pathologists clinical practice guideline update. *J Clin Oncol*. 2013;31(31):3997–4013.

Supplemental Figures for

A predictive endocrine resistance index accurately stratifies luminal breast cancer treatment responders and non-responders

Guokun Zhang[1], Vindi Jurinovic[1], Stephan Bartels[2], Matthias Christgen[2], Henriette Christgen[2], Leonie Donata Kandt[2], Lidiya Mishieva[3,4], Hua Ni[5], Mieke Raap[2], Janin Klein[6], Anna-Lena Katzke[6,] Winfried Hofmann[6], Doris Steinemann[6], Ronald E. Kates[7], Oleg Gluz[7,8,9], Monika Graeser[7,8,10], Sherko Kümmel[7,11,12], Ulrike Nitz[7,8], Christoph Plass[13], Ulrich Lehmann[2], Christine zu Eulenburg[7,14], Ulrich Mansmann[1], Clarissa Gerhäuser[13*], Nadia Harbeck[5,7], Hans H. Kreipe[2,7]

[1]Institute for Medical Information Processing, Biometry, and Epidemiology, Medical Faculty, Ludwig Maximilians University (LMU), Munich, Germany
[2]Institute of Pathology, Hannover Medical School, Hannover, Germany
[3]Institute of Sociology (IfS), Faculty of Social Sciences, University of Bremen, Bremen, Germany
[4]Department of Methodology and Statistics, Faculty of Social & Behavioural Sciences, Utrecht University, Utrecht, The Netherlands
[5]Breast Center, Department OB&GYN and CCC Munich, LMU University Hospital, Munich, Germany
[6]Department of Human Genetics, Hannover Medical School, Hannover, Germany
[7]West German Study Group (WSG), Moenchengladbach, Germany
[8]Ev. Bethesda Hospital, Breast Center Niederrhein, Moenchengladbach, Germany
[9]University Clinics Cologne, Women's Clinic and Breast Center, Cologne, Germany
[10]Department of Gynecology, University Medical Center Hamburg, Germany
[11]Clinics Essen-Mitte, Breast Unit, Essen, Germany
[12]Charité - Universitätsmedizin Berlin, Department of Gynecology with Breast Center, Berlin, Germany
[13]Division of Cancer Epigenomics, German Cancer Research Center (DKFZ), Heidelberg, Germany
[14]Department of Biometry and Epidemiology, University Medical Center Hamburg, Hamburg, Germany

Present address HN: Department of Pathology, School of Basic Medical Sciences, Peking University Third Hospital, Peking University Health Science Center

*Corresponding author:
Dr. Clarissa Gerhäuser, Division of Cancer Epigenomics, German Cancer Research Center, Im Neuenheimer Feld 280, 69120 Heidelberg, Germany
Phone: +40 6221 423306, email: c.gerhauser@dkfz.de

Supplemental Figure 1: Endocrine therapy-induced changes in histological grade

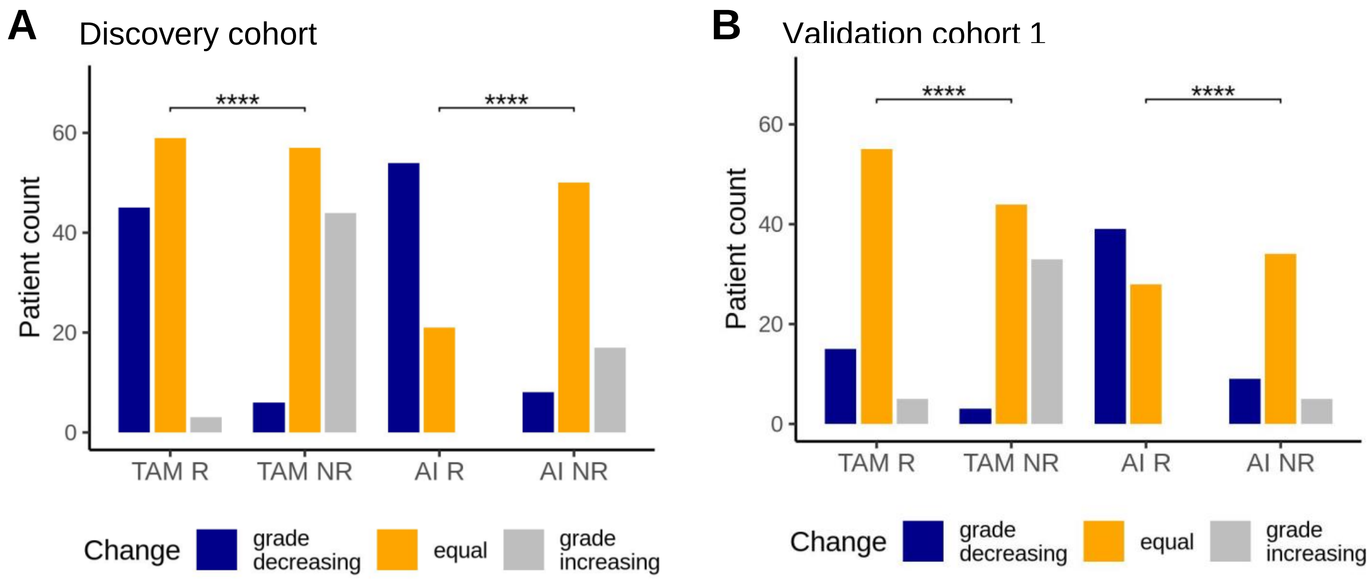

Supplemental Figure 1: Endocrine therapy-induced changes in histological grade per treatment group in the discovery cohort (N=364, TAM N=214, AI N=150) (**A**) and validation cohort 1 (N=270, TAM N=155, AI N=115) (**B**). Statistical differences tested using Chi-squared test with *P* value **** < 0.0001.

Supplemental Figure 2: Correlations of quantitative clinical features with TME components and PERCI scores

A

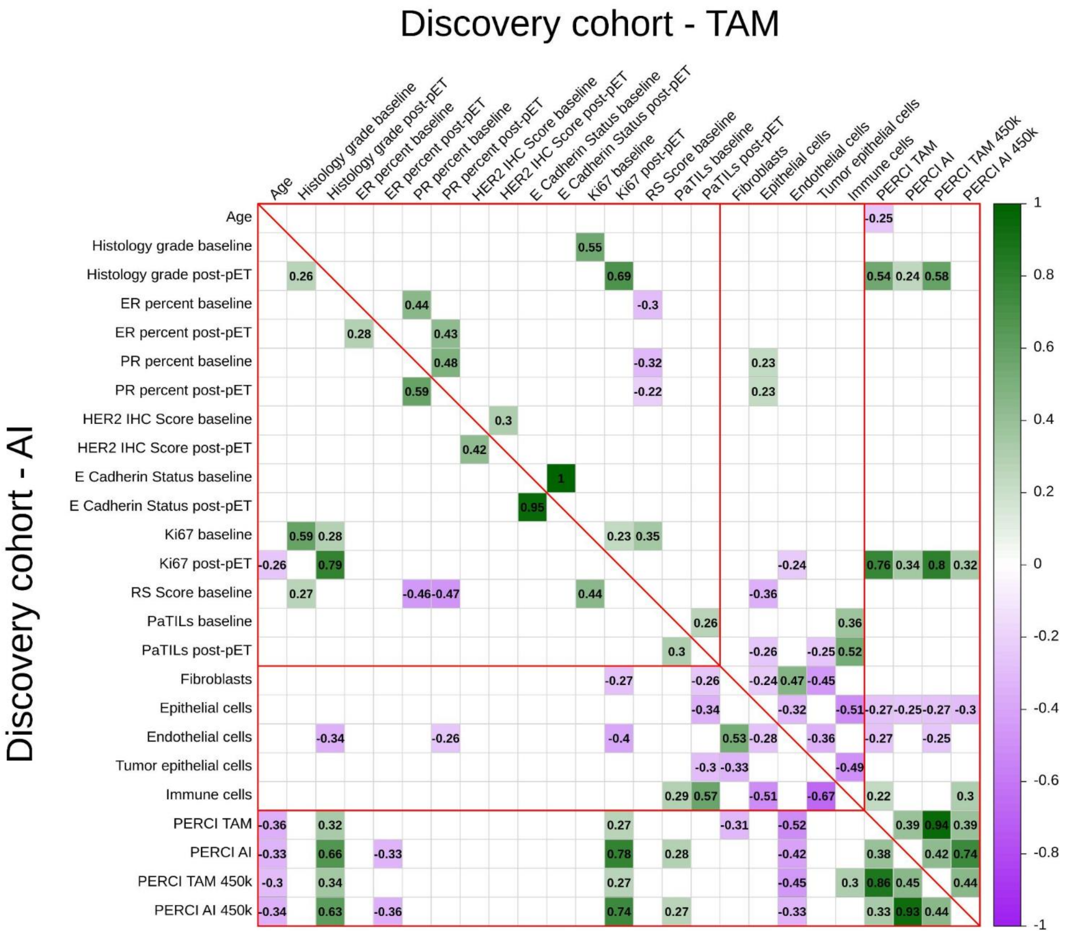

B

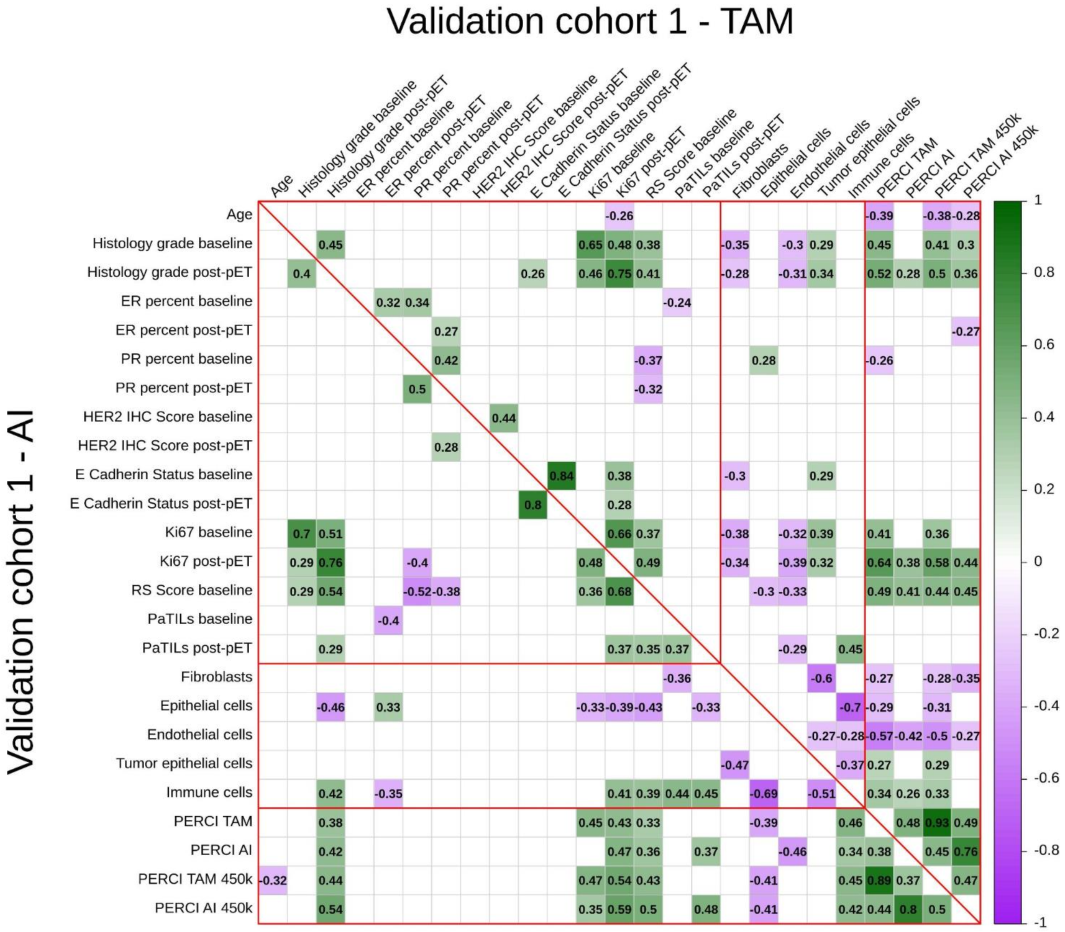

Supplemental Figure 2: Correlations of clinical features with TME components and PERCI scores. Spearman correlation coefficients were calculated between selected features in the discovery cohort (N=360, TAM N=210, AI N=150) (**A**) and validation cohort 1 (**B**) for the TAM and AI groups. Correlations are indicated by a color gradient from purple (-1) to green (1). Only results with statistically significant differences between response groups are shown (two-tailed test of significance which compares the observed value of correlation coefficient to its expected value under the null hypothesis (no correlation between the two variables), fdr-corrected *P* value < 0.01).

Supplemental Figure 3: Correlations of categorical clinical features with recurrent genomic alterations

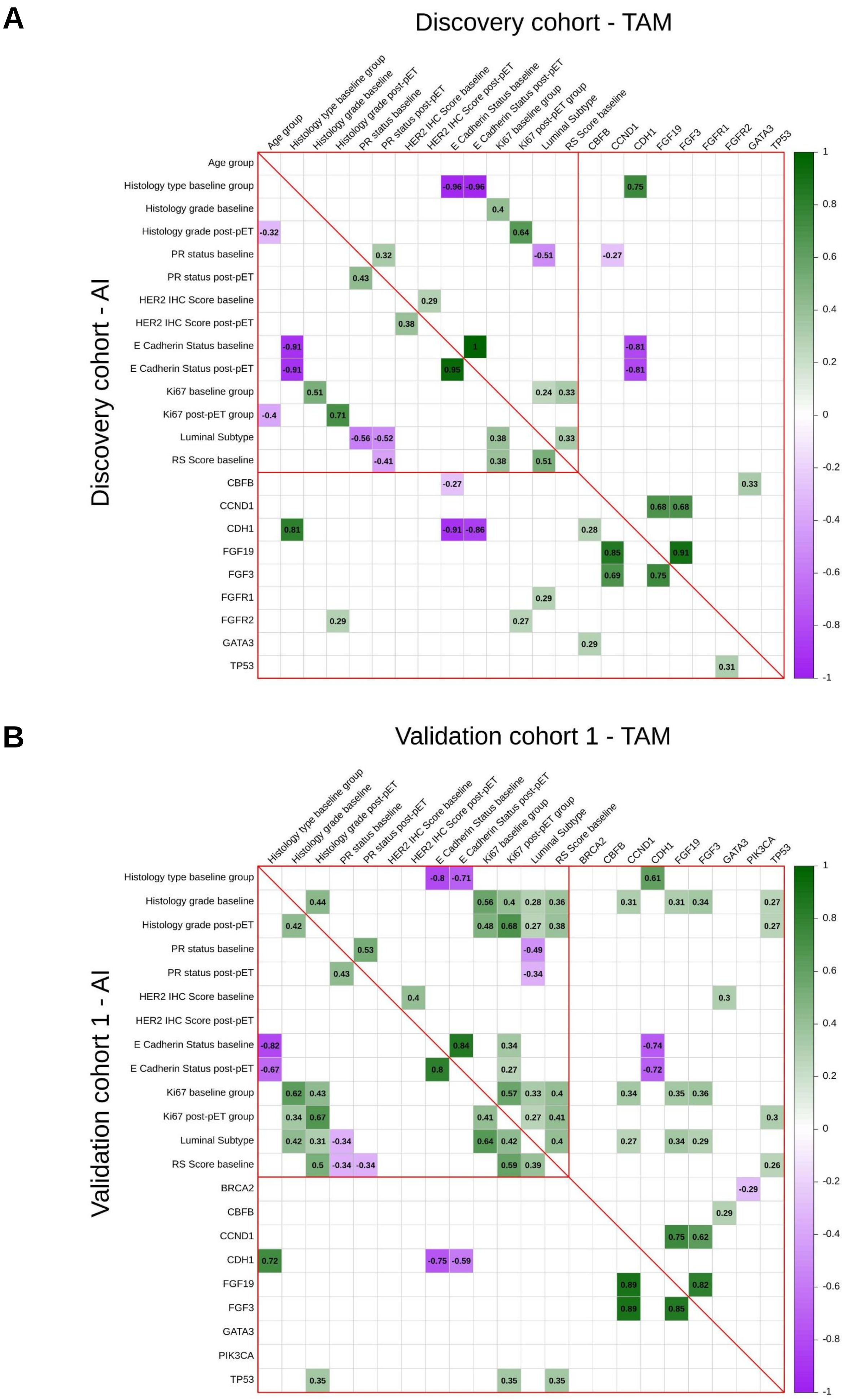

Supplemental Figure 3: Correlations of categorical clinical features with recurrent genomic alterations. Kendall correlation for ordinal clinical data and mutation counts in the discovery cohort (**A**) and validation cohort 1 (**B**) for the TAM and AI groups. Only results with statistically significant differences between response groups are shown (two-tailed test of significance which compares the observed value of correlation coefficient to its expected value under the null hypothesis (no correlation between the two variables), fdr-corrected *P* value < 0.01).

Supplemental Figure 4: pET resistance-related alterations in the methylome and gene expression.

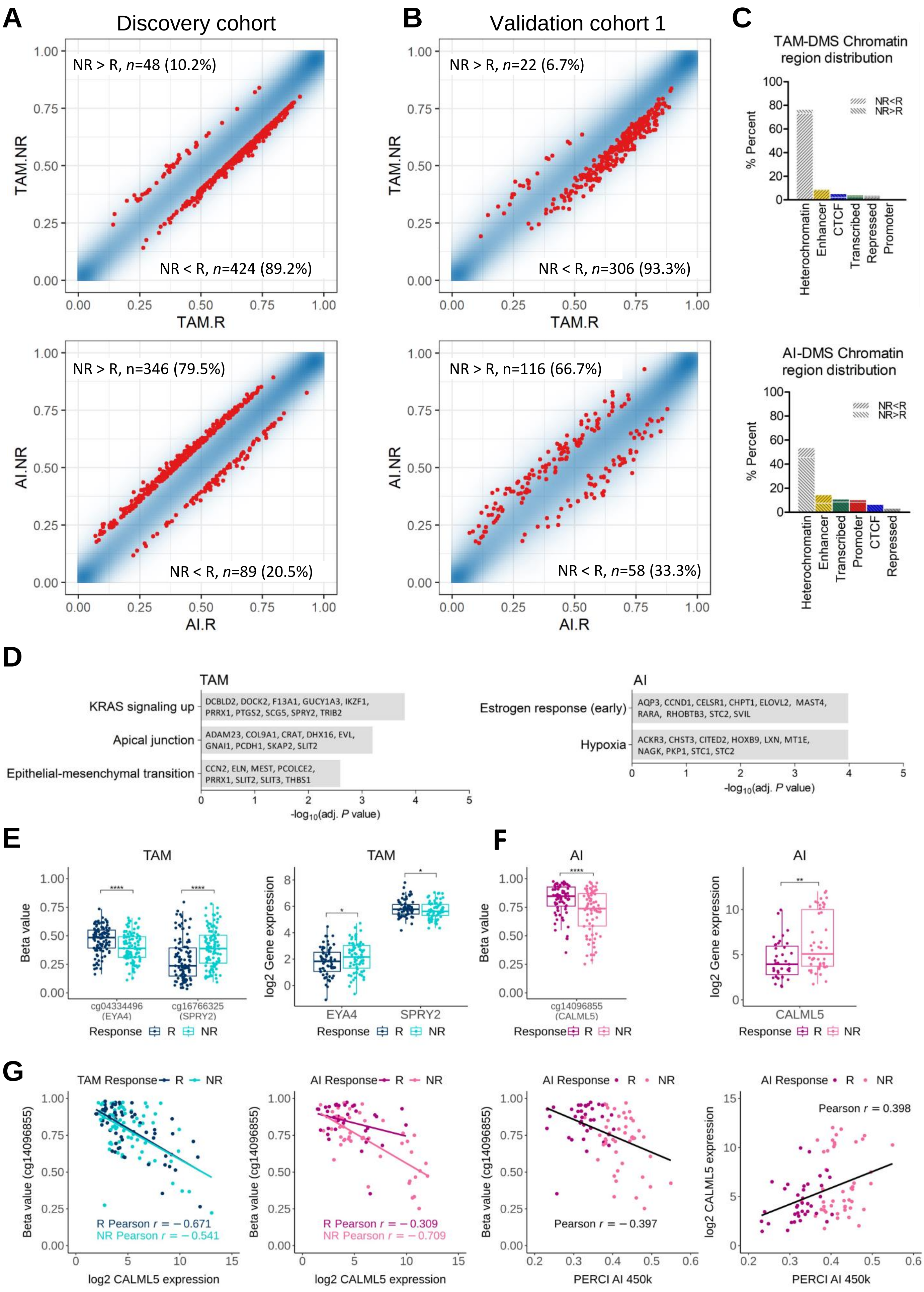

Supplemental Figure 4: pET resistance-related alterations in the methylome and gene expression (**A**) Density plots of mean methylation beta values of responder vs. non-responder groups (blue dots) for TAM- (upper panel) and AI-treated cases (lower panel) in the discovery cohort (*N* = 360, TAM *N* = 210, AI *N* = 150). Differentially methylated CpG sites (DMS) with a 10% methylation difference between R and NR ($p < 0.005$, limma) are highlighted in red (TAM: *n*=472 DMS, AI: *n*=435 DMS). Numbers of DMS with loss (NR < R) and gain in methylation in the NR group (NR > R) are indicated in the corners. (**B**) Density plots of mean methylation beta values of R vs. NR groups (blue dots) for TAM- (upper panel) and AI-treated cases (lower panel) in the validation cohort 1 (*N* = 222, TAM *N* = 131, AI *N* = 91). DMS from (**A**) with a 5% methylation difference between R and NR ($P<0.05$, limma) are highlighted in red (TAM: *n*=328 DMS, AI: *n*=174 DMS). (**C**) Percentage of DMS, split in hypo- and hypermethylated in NR, overlapping with chromatin regions derived from ChromHMM analyses of MCF7 cells (upper: TAM, lower: AI). (**D**) Gene set overrepresentation analysis of genes associated with TAM DMS (upper) and AI DMS (lower) in Molecular Signatures Database hallmark gene sets (with FDR-adjusted *P* value<0.005). (**E,F**) Expression of genes with significant association between DMS beta values and gene expression in a subset of the discovery cohort. Gene expression at baseline was determined using Nanostring BC360 arrays (*N* = 220, TAM *N* = 135, AI *N* = 85). Significant differences in methylation and mRNA expression between response groups were analyzed using student's t-test. (**G**) Pearson's correlation coefficients were calculated between cg14096855 methylation beta values and log2 *CALML5* mRNA expression values for both TAM and AI treatment (two left panels) as well as between cg14096855 methylation beta values or log2 *CALML5* expression and PERCI 450k for the AI group (two right panels). Boxplots show median (line), upper, and lower quartiles (boxes), and lines extending to 1.5-IQR (whiskers).

Supplemental Figure 5: pET resistance-related alterations in the tumor microenvironment.

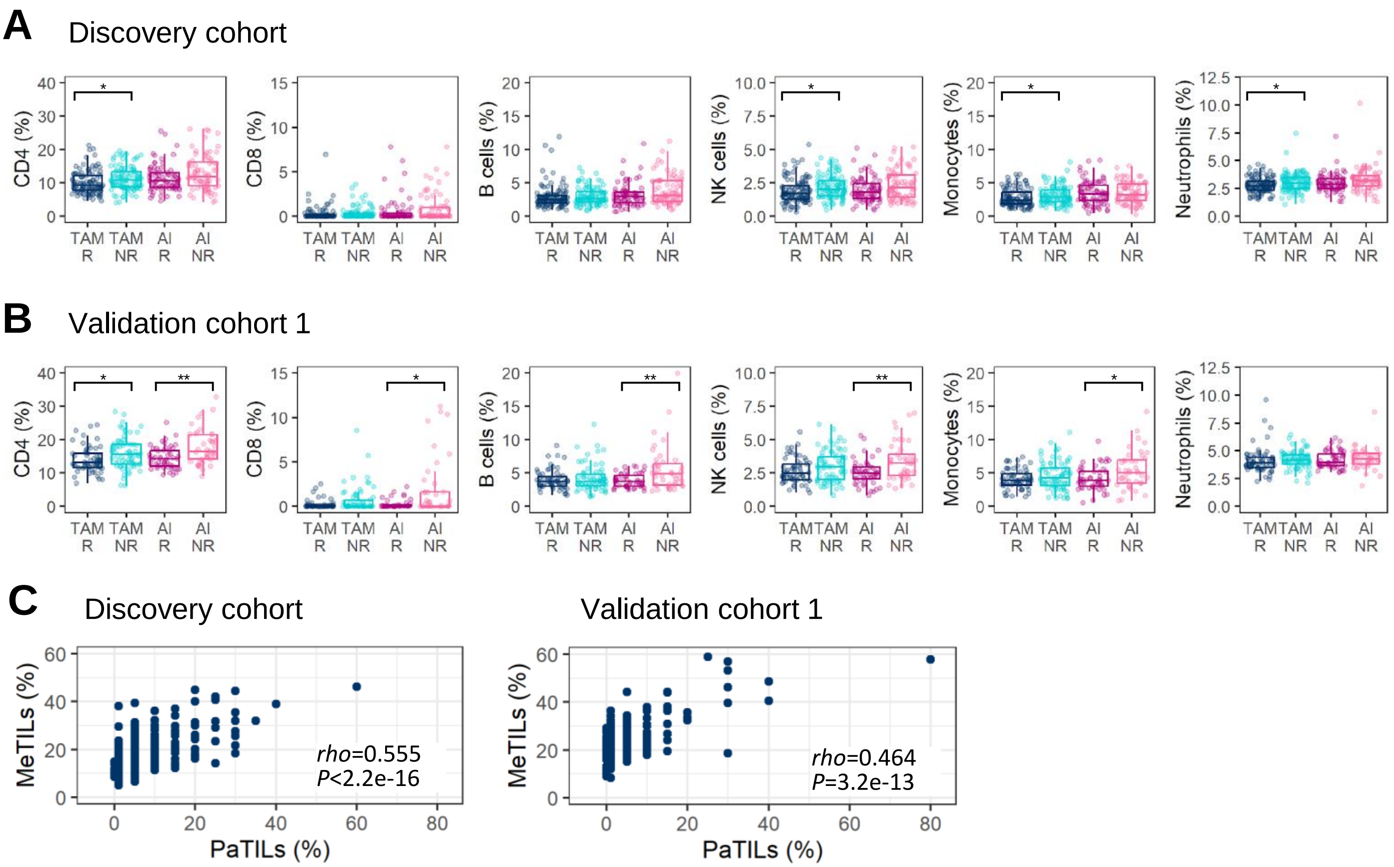

Supplemental Figure 5: pET resistance-related alterations in the tumor microenvironment. Boxplots of major immune cell fractions calculated from methylation reference datasets using the Houseman algorithm (Methods) in the discovery (*N* = 360, TAM *N* = 210, AI *N* = 150) (**A**) and validation cohort 1 (*N* = 222, TAM *N* = 131, AI *N* = 91) (**B**). R and NR groups per pET were compared using Wilcoxon test with *, **, *** FDR-adjusted *P* value < 0.05, 0.01, 0.001. Boxplots show median (line), upper, and lower quartiles (boxes), and lines extending to 1.5-IQR (whiskers). (**C**) Scatter plots depicting percentage of stromal TILs quantified by visual assessment on routine H&E-stained slides (PaTILs) vs. percentage of methylation-derived TILs (MeTILs, sum of CD4+, CD8+, B cell and NK cell percentages). Spearman's rank correlation coefficient *rho* and *P* value for the correlation between both methods is indicated.

Supplemental Figure 6: ROC-AUC of all predictors of PERCI TAM.

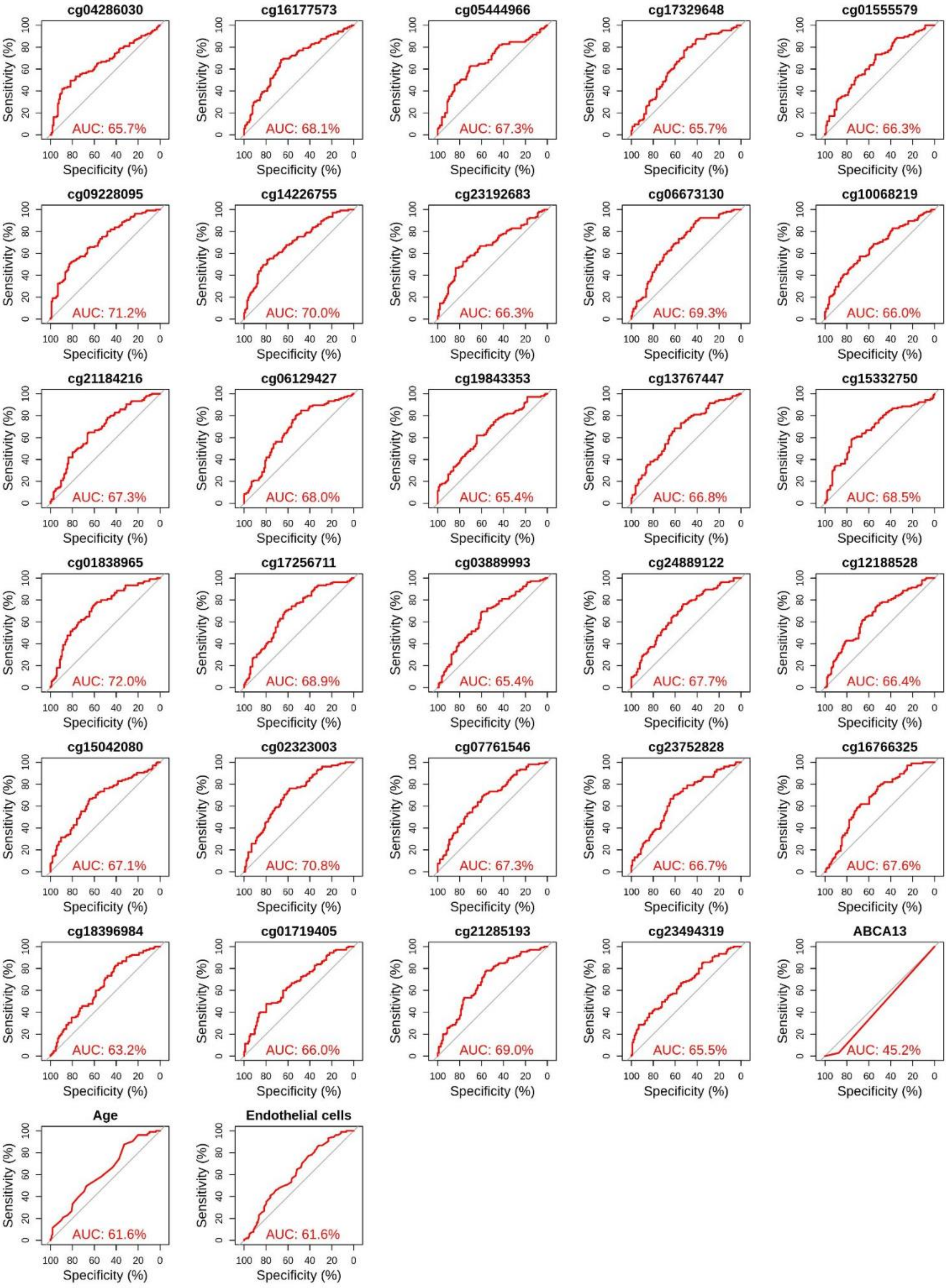

Supplemental Figure 6: ROC-AUC of all predictors of PERCI TAM. ROC-AUC of the individual predictors of PERCI TAM in the TAM discovery cohort (N = 360, TAM N = 210, AI N = 150). The x-axis shows the specificity, while the y-axis shows the sensitivity.

Supplemental Figure 7: ROC-AUC of all predictors of PERCI AI.

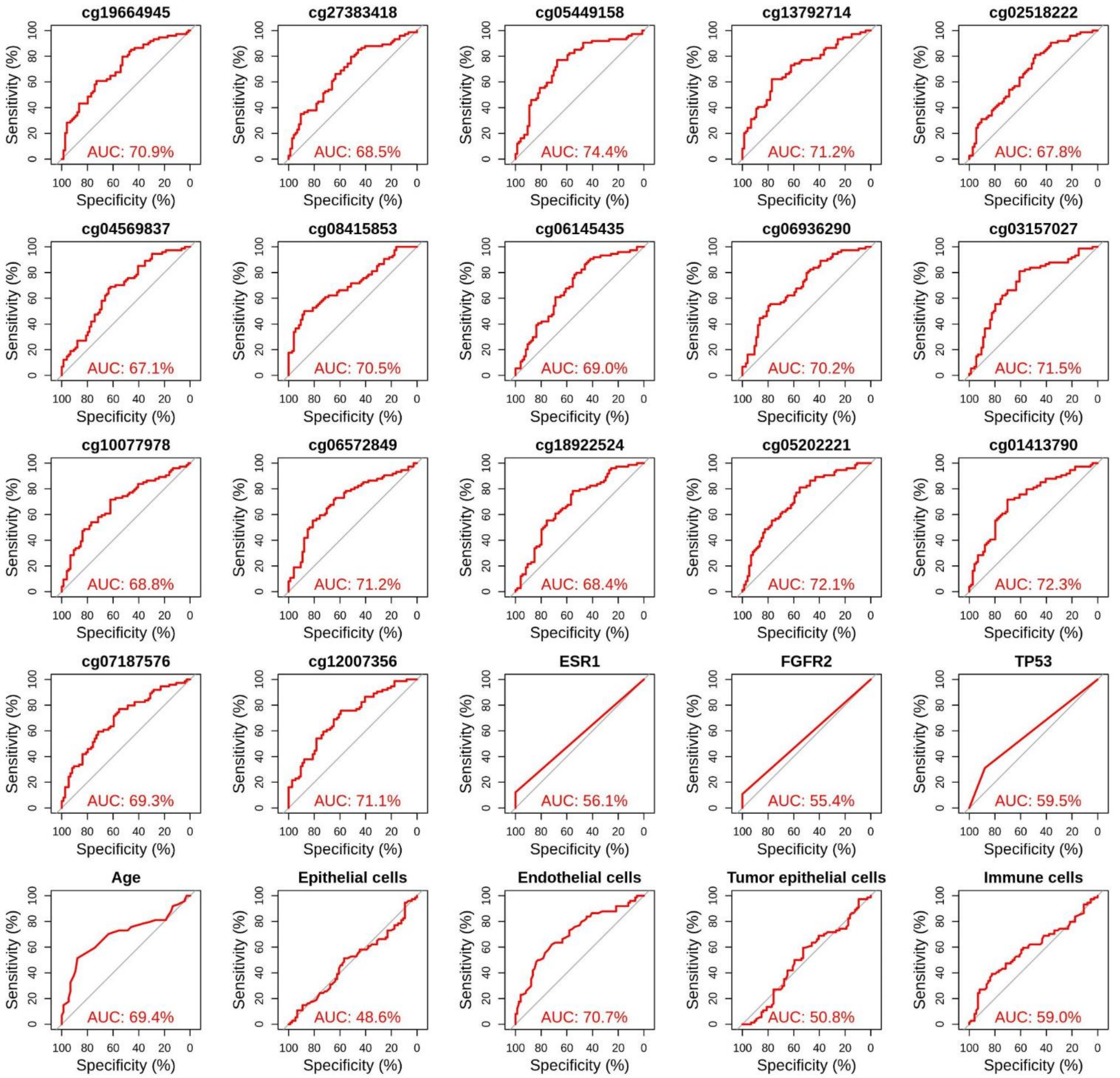

Supplemental Figure 7: ROC-AUC of all predictors of PERCI AI. ROC-AUC of the individual predictors of PERCI AI in the AI discovery cohort (*N* = 360, TAM *N* = 210, AI *N* = 150). The x-axis shows the specificity, while the y-axis shows the sensitivity.

Supplemental Figure 8: Performance of PERCI TAM 450k and PERCI AI 450k in the discovery and validation cohort 1.

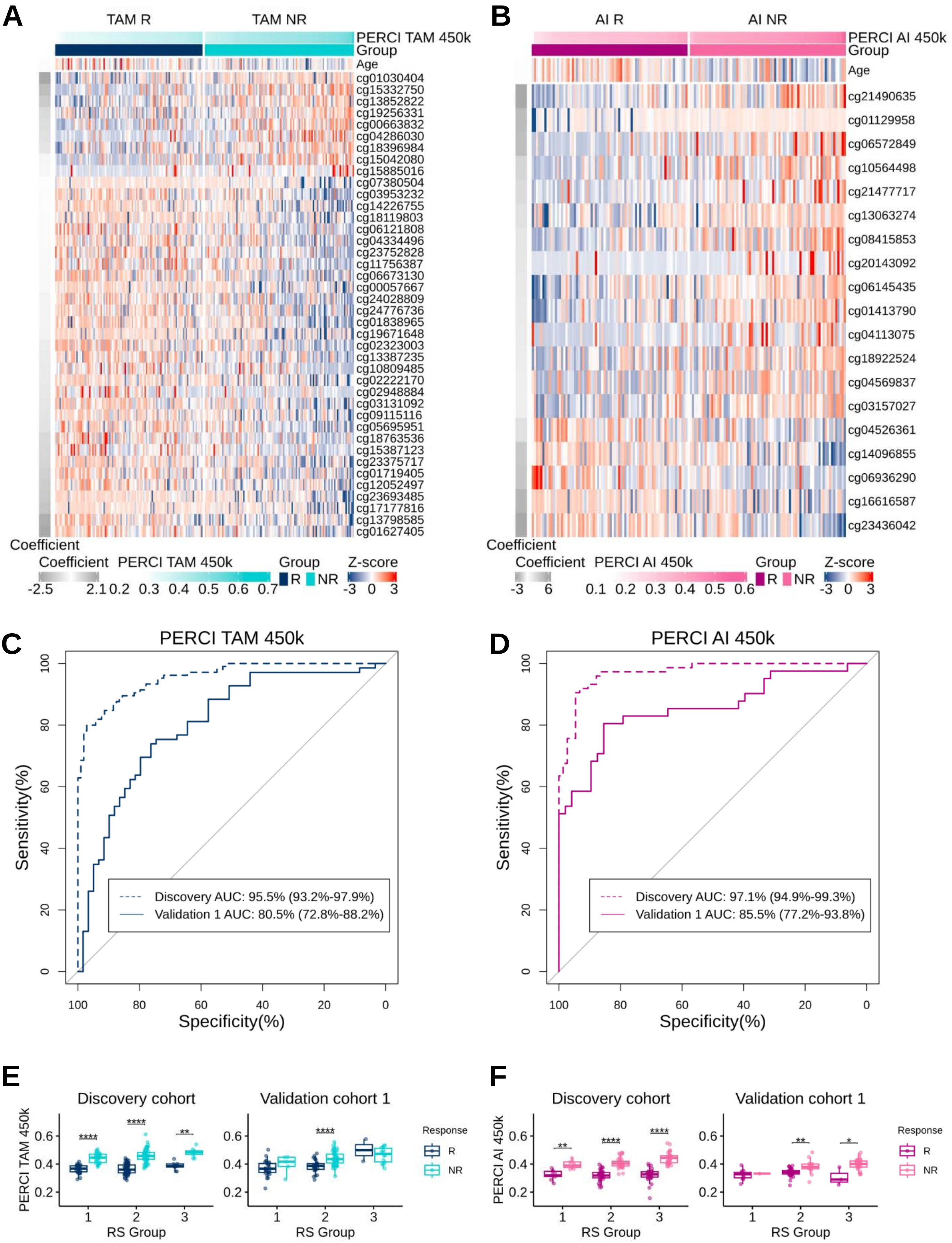

Supplemental Figure 8: Performance of PERCI TAM 450k and PERCI AI 450k in the discovery and validation cohort 1. Heatmap of methylation z-scores of age and selected CpG sites to build PERCI TAM 450k (**A**) and PERCI AI 450k (**B**). Analysis of the performance of PERCI 450k (**C**: TAM; **D**: AI) in the discovery cohort (N = 357, TAM N = 209, AI N = 148) and validation cohort 1 (N = 217, TAM N = 128, AI N = 89) by ROC-AUC. The x-axis shows specificity and the y-axis shows sensitivity. AUC with 95% CI. (**E-F**) Performance of PERCI TAM 450k and PERCI AI 450k, stratified by RS subgroups in the discovery cohort (N = 357, TAM N = 209, AI N = 148) (**E**) and the validation cohort 1 (N = 217, TAM N = 128, AI N = 89) (**F**). R and NR groups were compared using (paired, **E**) Wilcoxon test with *, **, ***, **** FDR-adjusted P value < 0.05, 0.01, 0.001, 0.0001.

Supplemental Figure 9: Recurrent genomic alterations (RGA) in the TCGA BRCA sub-cohort.

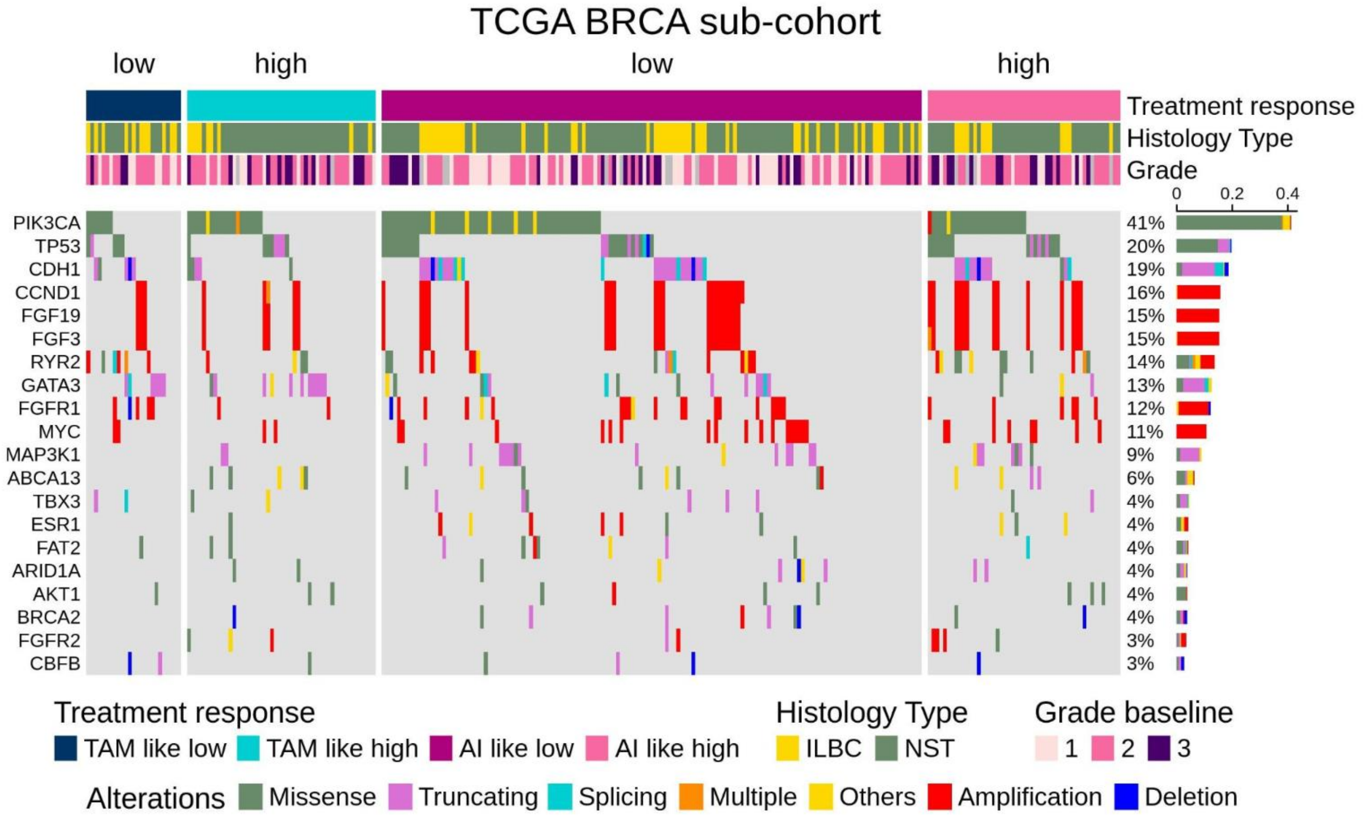

Supplemental Figure 9: RGA in the TCGA BRCA sub-cohort. The oncoprint summarizes the mutational landscape in the TCGA-BRCA sub-cohort (N=269, TAM-like N=75, AI-like N=194), color-coded by the mutational type and separated into high and low PERCI 450k, sorted by total alteration burden. Genes were selected based on Figure 3A. Clinical annotations of cases are indicated on the top. The barplot at the right quantifies the recurrence of the RGA.
